# From batch to flow plasmon catalysis: revealing mass transport limits in Au@Pd nanocatalysts for Suzuki coupling

**DOI:** 10.1039/d5nr03832d

**Published:** 2025-12-12

**Authors:** Mariia Erzina, Daria Votkina, Elena Miliutina, Oleg Gorin, Malek Y. S. Ibrahim, David M. Köpfler, Tobias Friedl, Christian Koller, Junais Habeeb Mokkath, Mufasila Mumthaz Muhammed, Markus Valtiner, Oleksiy Lyutakov, Olga Guselnikova

**Affiliations:** a Department of Solid-State Engineering, University of Chemistry and Technology Technicka 5 Prague 166 28 Czech Republic; b Research School of Chemistry and Applied Biomedical Sciences, Tomsk Polytechnic University Lenina Avn. 30 Tomsk 634050 Russian Federation; c Redeem Solar Technologies GmbH Großmarktstraße 8a 8020 Graz Austria; d University of Applied Sciences Wiener Neustadt Johannes Gutenberg-Straße 3 2700 Wiener Neustadt Austria; e Fotec – Forschungs- und Technologietransfer GmbH, Wiener Neustadt Viktor Kaplan-Straße 22700 Wiener Neustadt Austria; f College of Integrative Studies, Abdullah Al Salem University Khaldiya Campus Kuwait; g College of Engineering, International University of Science and Technology in Kuwait Ardiya Kuwait; h Vienna University of Technology, Institute of Applied Physics Karlsplatz 13 1040 Vienna Austria olga.guselnikova@tuwien.ac.at

## Abstract

Plasmonic catalysis, as a powerful tool for synthetic transformations, has the potential to impact wide-scale applications by converting solar light into energy for chemical reactions. Current studies are limited to mL-scale batch reactors with mg-level nanocatalysts, lacking feasibility in common laboratory and industrial configurations. To overcome this limitation, transition of plasmonic chemistry from batch to flow mode is foreseen; however, there is a lack of understanding of how plasmon-driven processes couple with mass transport. To address this issue, we designed plasmonic catalysts for a flow system at a tens of mL scale employing gram-scale Au@PdNPs–Al_2_O_3_ nanostructures in a flow reactor. Using Suzuki cross-coupling as a model reaction, we showed that the flow mode for Au@PdNPs–Al_2_O_3_ increases the reaction rate, the time to full conversion and the apparent quantum yield (AQY) ×3 compared to batch mode and outperforms previously reported examples/cases. Fluid dynamic simulations showed the critical effect of the residence times of nanocatalyst–reactant complexes under illumination on the product yield. This was consistent with photocurrent measurements, revealing that the electron transfer efficiency is enhanced under increased mass transport conditions. Unlike previous studies that primarily emphasized the carrier dynamics within metal–metal/semiconductor heterojunctions (*e.g.*, Au/Pd) in batch mode, our flow system demonstrates that efficient carrier transfer to reactants is critical for achieving high TONs and AQYs. This work provides the first framework for translating plasmonic catalysis into flow, offering design principles for future light-driven chemical processes beyond conventional batch mode.

## Abstract 2

Transitioning plasmonic chemistry from batch to flow, we show that controlled mass transport in a flow reactor unlocks hot-carrier transfer in gram-scale Au@Pd–Al_2_O_3_ catalysts, tripling Suzuki coupling and enabling scalable light-driven synthesis.

## Graphical abstract

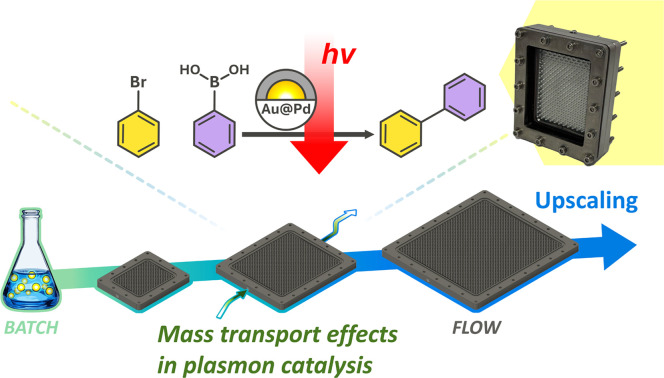

## Introduction

Plasmon catalysis using low-energy-consuming nanostructures is a powerful tool for synthetic transformations,^1,2^ which has the potential to impact industrial applications.^3,4^ It enables the transfer of energy from nanoparticles to molecules through non-thermal mechanisms like hot carrier movement and intramolecular electron excitation, as well as through thermal effects like plasmonic heating.^5,6^ The main appeal of plasmon-assisted chemistry lies in developing low-cost, sustainable technologies that harness solar energy to produce high-value chemicals.^7,8^ Recent advances focus on designing plasmonic catalysts^9^ that enhance photocatalytic efficiency through synergy between active sites and plasmonic excitation.^2,10^ For instance, plasmon-enhanced photochemical ethylene oxide production has been explored as an industrially relevant process,^11^ while plasmon-driven azide–alkyne cycloaddition has been demonstrated to be a sustainable approach for copper-free click chemistry.^12,13^ At the same time, Suzuki cross-coupling reactions have been developed as a key strategy for carbon–carbon bond formation in industrial and pharmaceutical applications.^14,15^ The Suzuki reaction has also been probed under plasmonic catalytic conditions.^16–22^

One of the major bottlenecks in plasmonic catalysis is the lack of scalability demonstrations, as most studies remain limited to small-scale trials without addressing scale-up.^1,3,8^ Most of the recent reports on plasmon catalysis for organic transformations utilize a reaction volume of a few mL in various configurations of batch mode reactors using mg-scale dispersed plasmonic nanocatalysts. Currently, there is a lack^23,24^ of quantitative feasibility studies on plasmonic catalytic processes in a relevant industrial, or at least close to industrial, configuration. However, the challenge here is beyond technological scale-up but lies in a fundamental issue – the inefficient mass transport at the solid (nanocatalyst)–liquid interface in batch mode. Plasmon-enhanced reactions often rely on localized hotspots, leading to severe diffusion limitations. These mass transport constraints arise due to the inefficient diffusion of reactants to plasmonic hotspots, particularly in high photon flux environments, where reactants are depleted faster than they are replenished from the bulk solution. This phenomenon creates a diffusion-controlled regime as recently suggested by Alberto Naldoni,^25^ ultimately leading to limited reaction rates despite the availability of hot carriers.

In order to overcome this limitation, the challenge could be broken down to probing the relevant reactor configuration and relevant plasmonic nanocatalyst synthesis. The industrial configuration for plasmon catalysis could be borrowed from the classic photocatalysis setup,^26,27^ where there are some examples, such as the synthesis of vitamin D, caprolactam, and rose oxide, the latter estimated at the tons level annually.^28^ For example, Can Li developed a direct solar array-type flat-plate photochemical reaction system with a reaction area of 5 m^2^ with PerfectLight company.^29^ Thus, one could attempt to apply the lessons learned on mechanistic studies^27,30^ to upscaled plasmonic nanosystems.

As an alternative solution for plasmon catalysis, plasmon-driven chemistry is tested in continuous-flow reactors using Au@Pd-based plasmonic catalysts. We probed plasmon-driven Suzuki-cross coupling as a representative example of one of the scenarios for plasmonic chemistry. For flow reactor experiments, we prepared g-scale plasmonic nanoparticles based on commercial Al_2_O_3_*via* microwave-assisted AuNP growth and thin Pd layer reduction. These Au@PdNPs–Al_2_O_3_ samples were used in tens of mL volumes for plasmonic Suzuki coupling. We hypothesize that mass transfer processes governing carrier delivery to reactants are critical for efficient photon utilization, a factor largely overlooked in previous plasmonic studies. By integrating lessons from plasmonic reaction kinetics, photon penetration, and mass transport limitations, upscaled plasmonic experiments were tailored to maximize the benefits of plasmonic energy conversion in flow mode.

## Results and discussion

### Gram-scale synthesis of plasmonic catalysts

**Fig. 1:**
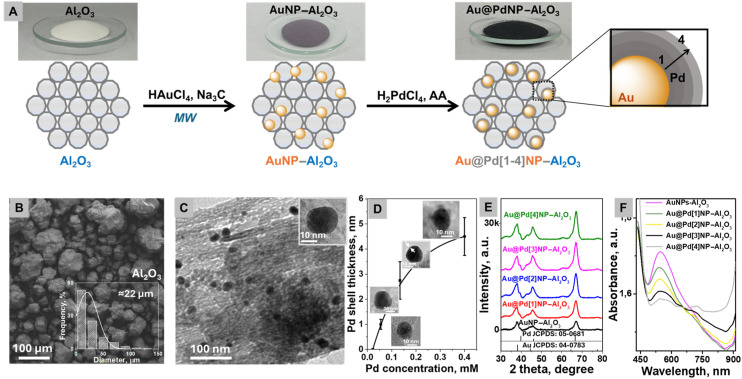
(A) Fabrication of Au@PdNPs–Al_2_O_3_ on a gram scale, (B) SEM image of pristine Al_2_O_3_ (insert: size distribution), (C) TEM image of AuNPs–Al_2_O_3_, (D) Pd shell thickness against the Pd concentration (the error bars indicate the standard deviation from ∼100 particles per sample), (E) XRD patterns of the prepared AuNPs–Al_2_O_3_ and Au@Pd[1–4]NPs–Al_2_O_3_, and (F) UV-vis spectra of the prepared AuNPs–Al_2_O_3_ and Au@Pd[1–4]NPs–Al_2_O_3_.

We commenced our investigation with the synthesis of plasmonic catalysts based on commercially available Al_2_O_3_ porous materials (0.58 nm pore size and 22 ± 1.5 µm particle size) that served as a support for plasmon-active AuNPs deposition (Fig. 1A and B). The high surface area materials provided a platform for the deposition of plasmonic NPs.^31^

AuNPs were deposited on Al_2_O_3_ by a microwave assisted approach^32^ using sodium citrate as a reducing agent (Fig. S1). In contrast to common complex multi-step synthesis routes,^9^ this straightforward microwave-assisted technique is highly promising for inter-laboratory testing, thus shortening the timeline for the new technique to have a broad impact.^7^ 20 ± 3 nm-sized gold nanoparticles deposited on the Al_2_O_3_ surface exhibited a spherical morphology (Fig. 1C). The next step of Pd deposition was also realized on a gram scale *via* the reduction of H_2_PdCl_4_ with ascorbic acid.^33,34^

We prepared a set of Au@Pd[1–4]NPs–Al_2_O_3_ samples *via* addition of different amounts of the Pd precursor and characterized them by transmission electron microscopy (TEM) (Fig. 1D and Fig. S2), X-ray diffraction (XRD) (Fig. 1E), UV-vis spectroscopy (UV-vis) (Fig. 1F) and atomic absorption spectrophotometry (AAS) (Table S1). The TEM image of prepared AuNPs–Al_2_O_3_ shows the formation of spherical nanoparticles (Fig. 1C). After Pd precipitation on the surface of AuNPs–Al_2_O_3_, the dependence of Pd shell thickness on the Pd concentration (H_2_PdCl_4_) of Au@Pd[1–4]NPs–Al_2_O_3_ was analysed (evaluated from a gradual increase of Au@Pd size, estimated from TEM images). The dependence shows a nonlinear increase in shell thickness with increasing Pd concentration, indicating a saturation-like growth (Fig. 1D and Fig. S2). With an increase in the amount of Pd, the average diameter of Au@PdNPs deposited on Al_2_O_3_ nanoparticles increases from 20 ± 2 nm for Au@Pd[1]NPs–Al_2_O_3_ to 30 ± 3 nm for Au@Pd[4]NPs–Al_2_O_3_, indicating the growth of the Pd shell on the surface of AuNPs–Al_2_O_3_, confirmed by AAS (Fig. 1D and Table S1). The X-ray diffraction (XRD) pattern of the prepared Au@Pd–Al_2_O_3_ sample is shown in Fig. 1E. It is evident that the sample contains face-centered cubic Pd because peaks at 40.5°, 46.8°, and 68.4°, corresponding to the (111), (200), and (220) lattice planes, are observed (JCPDS card no. 05-0681) (Fig. 1E). So, it is clear that most of the strong diffraction peaks of Pd almost overlap with the peaks of Au at 38.2°, 44.9° and 64.6°, which correspond to the (111), (200) and (220) planes (JCPDS card no. 05-04783).^35,36^

The set of prepared materials Au@Pd[1–4]NPs–Al_2_O_3_ was characterized by UV-vis absorption spectroscopy (Fig. 1F). The absorbances of the catalyst and pristine AuNPs–Al_2_O_3_ were recorded in a water/ethanol solution to probe the plasmonic response under the same liquid-phase conditions as used for catalysis. This approach enables us to capture solvent- and interface-dependent spectral shifts.^37^The last sample has a well-recognized absorbance peak at 550 nm that corresponds to the plasmon resonance of spherical gold nanoparticles with 20 nm diameter loaded onto Al_2_O_3_. Deposition of the Pd layer in the catalyst system led to a decrease in the absorbance of AuNPs and the rise of a new absorbance maximum in the ∼700 nm region that could be attributed to hybridization at the Au–Pd interface that covers Au.^38^

### Probing Pd shell thickness towards maximum catalytic performance (batch mode) *via* experimental and theoretical approaches

**Fig. 2:**
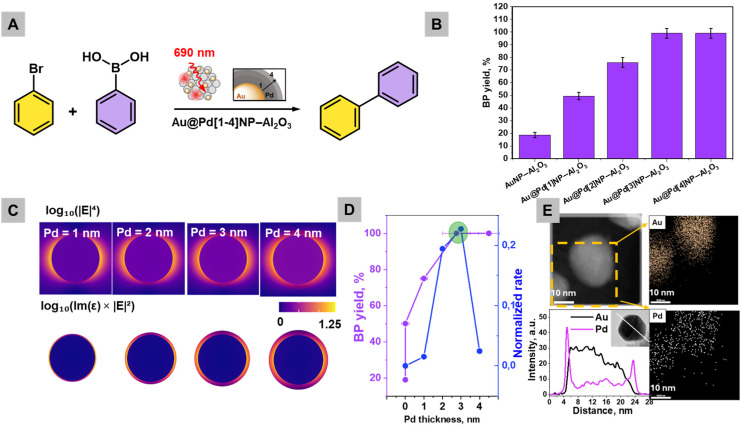
(A) Scheme of Suzuki cross-coupling using Au@Pd[1–4]NPs–Al_2_O_3_, (B) BP analytical yield (GC measured) in 4 hours of reaction using Au@Pd[1–4]NPs–Al_2_O_3_ (the yields are means and the error bars represent the standard deviation from three independent measurements (*n* = 3)), (C) simulated near-field electromagnetic enhancement (top row) and hot carrier generation maps (bottom row) for single Au@Pd nanoparticles with a 20 nm Au core and varying Pd shell thicknesses (1–4 nm), (D) correlation between Pd shell thickness (20 nm Au core), experimental catalytic performance (BP yield after 4 hours of reaction), and simulated Suzuki coupling activity, and (E) STEM-EDX mapping images of an individual nanoparticle of Au@PdNPs–Al_2_O_3_ and its elemental line profile.

Based on UV-vis spectra, we chose an LED with an excitation wavelength of 690 nm for the further catalytic experiments (Fig. S3). Longer wavelengths (>600 nm) could penetrate more deeply into the reaction media, providing a more uniform activation of photocatalysts and substrates. This is especially beneficial for large-scale industrial applications.^3^ The prepared Au@Pd[1–4]NPs–Al_2_O_3_ samples were tested in the Suzuki coupling reaction between bromobenzene and phenylboronic acid under light irradiation at 690 nm (300 mW cm^−2^) in batch mode with a reaction volume of 20 mL (Fig. 2A and B). The analytical yield of biphenyl was verified by gas chromatography (GC) after 4 hours of reaction with preliminary calibration for quantitative analysis (Fig. S4 and 5). In Fig. 2B, AuNPs–Al_2_O_3_ show minimal catalytic activity, while the introduction of Pd doubles the product yield (Fig. 2B). However, increasing the thickness of the Pd shell beyond 3 nm (Au@Pd[3]NPs–Al_2_O_3_) does not lead to further catalytic performance improvement.

To explain the experimentally determined optimal Pd thickness of Au@Pd[3]NPs–Al_2_O_3_, simulations of electromagnetic field (EM) enhancement and hot electron generation rates were performed (Fig. 2C). First, the absorption cross-section of Au@Pd with increasing Pd shell thicknesses was calculated using the *T*-matrix method (Fig. S6),^39^ which solves Maxwell's equations for spherical multilayered particles by decomposing the fields into vector spherical harmonics. The slight discrepancy between Fig. S6 and Fig. 1F is explained by neglecting the Al_2_O_3_ support and solvent interface effects. However, a red shift is observed with the Pd shell growing that is similar to the experimental UV-vis spectra shown in Fig. 1F. Upon coating with Pd, the absorption spectra exhibit a progressive red shift with the increase of thickness from 1 to 4 nm that occurs due to the Pd shell's significant plasmon damping and dielectric screening.^40^Fig. 2C (upper row) presents the simulated Raman enhancement factor (EF), approximated as log_10_(|*E*|^4^). The enhancement maps reveal that near-field hotspots are initially localized at the outer shell, but both their intensity and spatial extent diminish as the Pd shell thickness increases due to the increased absorption and suppression of LSPR by Pd.^40^

The simulated hot carrier distribution, expressed as log_10_(Im(*ε*) × |*E*|^2^), serves as a qualitative indicator of the local generation rate of hot electrons and holes driven by plasmon-induced intraband and interband transitions^41^ (Fig. 2C, lower row), with Im(*ε*) representing the imaginary part of the local dielectric function and |*E*|^2^ reflecting the local field intensity enhancement. Simulated hot carrier distributions reveal that carrier generation becomes increasingly concentrated at the Au–Pd interface and the Pd surface with thicker shells, owing to Pd's high Im(*ε*).^42,43^ This indicates that Pd acts as a plasmonic energy sink, enabling localized carrier excitation. With all these results in hand, the rate profiles for the Au@PdNPs–Al_2_O_3_ nanoparticles were simulated and they exhibited a clear maximum in catalytic activity at a Pd shell thickness of ∼3 nm for a fixed 20 nm Au core (Fig. 2D). Simulated Suzuki activity (Fig. 2D) refers to a calculated relative rate constant derived from an Arrhenius-type kinetic model where the activation barrier is modulated by both hot-carrier flux decay through the Pd shell and plasmonic absorption enhancement. The observed trends are consistent with previous studies such as those by Christopher *et al.*^42^ and Baffou *et al.*,^43^ who demonstrated that plasmonic–catalytic hybrid structures generate hot carriers primarily in regions with high loss (Im(*ε*)) and field overlap. This trend arises from a balance between the hot-carrier flux and plasmonic absorption, suggesting an optimal geometry where the shell is thick enough to provide a catalytically active amount of Pd but thin enough to permit efficient hot-carrier transfer from the Au core.

Therefore, experimental and theoretical studies showed that Au@Pd[3]NPs–Al_2_O_3_ (abbreviated as Au@PdNPs–Al_2_O_3_) with ∼3 nm Pd thickness is optimal for the further catalytic studies (Fig. 2D). Notably, due to the efficient light-induced charge separation and surface activation facilitated by the plasmonic core–shell configuration, only trace amounts of Pd (0.09 wt% according to AAS) were sufficient for successful Suzuki coupling. The time-resolved GC measurements of Suzuki coupling in batch mode show that after 11 h, a high yield of biphenyl is obtained (Fig. 2B). The best-performing Au@PdNPs–Al_2_O_3_ sample was additionally characterized by STEM-EDX analysis (Fig. 2E) of an individual Au@PdNP, clearly indicating the homogeneous distribution of both Au and Pd. The formation of a core–shell structure is further supported by the elemental line profile (Fig. 2E) obtained from a selected single particle in STEM mode. The experimentally measured 2.7 ± 0.7 nm thickness of Au@PdNPs–Al_2_O_3_ (estimated as the width of two concentration peaks, located at the nanoparticle edges and corresponding to the Pd shell) slightly deviates from the theoretically predicted optimal thickness of 3 nm. However, the overall trends in Fig. 2D for experimentally measured and simulated thicknesses correspond to each other. Additionally, X-ray photoelectron spectroscopy (XPS) analysis showed that Au@PdNPs–Al_2_O_3_ has a Pd surface concentration of only 0.6 at% (Fig. S7). The Au 4f spectrum in Fig. S7B shows a deconvoluted doublet with peaks at 87.8 eV and 84.3 eV that can be assigned to 4f_5/2_ and 4f_7/2_, respectively.^44^ The successful loading of palladium is evidenced by the Pd 3d spectrum (Fig. S7C): two fitted peaks positioned at 340.5 eV and 335.5 eV correspond to the Pd 3d_3/2_ and 3d_5/2_ spin–orbit levels of palladium (0),^45^ confirming that Pd is deposited in a non-oxidized form. This approach aligns with current demands to reduce reliance on scarce and expensive metals, while leveraging nanoscale engineering to maintain catalytic efficiency. Such a design is especially valuable in photocatalytic systems where plasmonic activation can compensate for the lower catalyst loading by enhancing local electric fields and surface reaction rates.

### Suzuki C–C coupling flow mode experimental setup and optimization

**Fig. 3:**
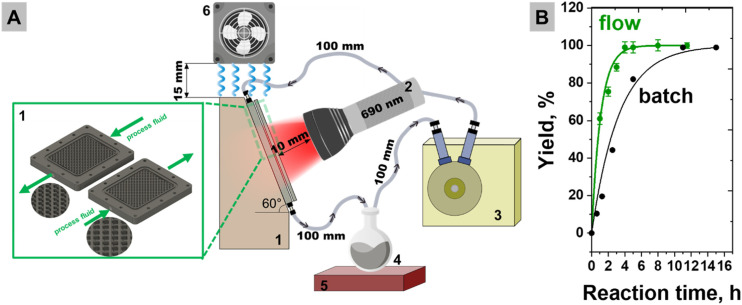
(A) Schematic illustration of the plasmonic flow reactor setup used for visible-light-driven Suzuki coupling reactions: (1) flow reactor, (2) 690 nm LED, (3) peristaltic pump, (4) stock flask, (5) magnetic stirrer, and (6) ventilator. (B) Kinetic curves of biphenyl formation obtained using Au@PdNPs–Al_2_O_3_ in batch mode and flow mode (optimized parameters of 60 mL min^−1^; the green arrows point upstream and backward flow) under 300 mW cm^−2^ irradiance (the yields are means and the error bars represent the standard deviation from three independent measurements (*n* = 3)).

A flat-plate, continuous-flow photoreactor (Arrow Reactor model) from Redeem Solar Technologies GmbH was employed in this study. The reactor is constructed from stainless steel and contains a rectangular flow channel (Fig. 3A). This channel design was chosen over a linear flow-through configuration to improve the light utilization and reaction efficiency. In a straight channel, laminar flow causes uneven photon penetration and limited mixing of catalyst particles. The arrow-shaped inserts disrupt this flow, generating recirculation zones and vortices that (i) extend the catalyst residence time in the illuminated zone,^46^ (ii) homogenize light exposure, and (iii) enhance the convective transport of reactants.^47^ We intentionally used a low-power LED (<1000 mW cm^−2^) to ensure that thermal effects (plasmonic heating) are minimized.^48^ Therefore, we expect the system to remain in a “purely photonic” regime, where any catalytic enhancement is due to hot charge carriers, not heat, to exclude heat-driven Suzuki coupling. Additionally, the usage of a cooler fan allowed us to keep a constant temperature of <30 °C during the plasmon-assisted reaction. The thermo-camera image shows insignificant heating of the reactor to a maximum temperature of 27.8 °C during irradiation (Fig. S8).

#### Experimental flow reactor set-up

The experimental set-up is presented in Fig. 3A (a photo is provided in Fig. S8) and contains (1) a photoreactor wall, containing the Au@PdNPs–Al_2_O_3_ suspension, (2) a 690 nm LED light source, with a 10 mm diameter beam directed at the catalytic surface, (3) a peristaltic pump that circulates the reaction mixture through the illuminated zone, (4 ) a reaction flask that contains the substrate mixture and serves as a reservoir for the liquid-phase reactants and products, (5) a magnetic stirrer, and (6) a ventilator. Initially, the reaction mixture (catalyst, reagents and solvents) was loaded into the stock flask equipped with a magnetic stir bar to minimize the precipitation of the catalyst.

Within the photochemical flow reactor, we repeated the same time-resolved measurements of Suzuki coupling of a total reaction volume of 20 mL (Fig. 3B). Samples were taken at regular time intervals from the collection vessel and the GC-measured analytical yield was plotted for all the reactors against the time of circulation. During circulation, the flow reactor was illuminated with the same LED (690 nm) and light irradiance was kept at a constant level (300 mW cm^−2^). The initial concentrations of the substrate, catalyst, and water were also kept constant for all the flow and batch experiments. Fig. 3B shows a comparison of the time-resolved biphenyl formation profiles for both the flow reactor and the batch (flask) experiments. The flow system shows a significantly steeper initial reaction rate (2.7 × 10^−4^ s^−1^ for flow and 1.05 × 10^−4^ s^−1^ for batch reactors, Fig. S9) and quantitative yield after 4 hours, highlighting the catalytic advantages of improved mass and photon transfer under flow conditions (Fig. S5 and Note S1). Therefore, further control experiments were also conducted for 4 hours. The control experiment in the dark and with illumination at 690 nm in the absence of NPs showed lower conversion and yield of 45% and <1%, respectively.

#### Experimental and computational fluid dynamics (CFD) analysis towards plasmonic reaction optimization

**Fig. 4:**
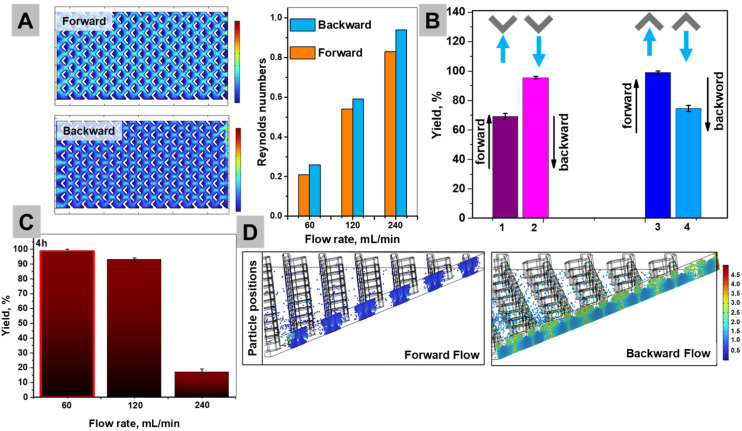
(A) CFD simulations of the Reynolds number of areas in cross-section for 60 ml min^−1^ of forward (top) and backward (bottom) flow rates. The column bars represent the summarized Reynolds numbers from different models. (B) Effect of flow (60 mL min^−1^) direction and arrow orientation on plasmon-driven Suzuki coupling (biphenyl yields obtained after 4 h of 690 nm illumination): (1) arrows point downstream and backward flow; (2) arrows point downstream and forward flow; (3) arrows point upstream and backward flow; and (4) arrows point upstream and forward flow (the yields are means and the error bars represent the standard deviation from three independent measurements (*n* = 3)). (C) Simulated positions of catalyst particles after 5 s at a flow rate of 60 mL min^−1^. The color indicates the residence time, with the brighter areas corresponding to longer exposure in the illuminated zone.

To optimize plasmon-enhanced catalysis, we combined experiments and CFD analysis on the tilted photoflow reactor (Fig. 4). The flow direction (forward *vs.* backward), arrow point (downstream *vs.* upstream), residence time, and convective mass transfer were systematically studied as potential parameters that can directly affect hot carrier utilization at the metal–reactant interface. The basic model parameters are shown in Fig. S10 and Note S2. While a full spatially resolved model coupling catalyst distribution, reactant concentration gradients, and photon absorption would provide deeper mechanistic insight, such an approach would be beyond the scope of this work. We envision that establishing such a “catalyst–reactant–photon” distribution model represents an important direction for future studies.

CFD simulations (Fig. S10–12) and experiments revealed that flow orientation (Fig. 4A and B) strongly influenced hydrodynamics and catalytic performance. At identical flow rates (300 rpm), the backward mode showed a higher average Reynolds number (Table S2) than the forward mode, indicating enhanced bulk mixing despite a slightly lower Re_max_ (9.89 *vs.* 12.9). Although both regimes remained laminar (Re < 2300), the improved convective flow in backward mode reduced mass transport limitations, leading to an ∼90% product yield *versus* ∼70% in forward flow (Table S2). Tracer simulations confirmed that gravitational effects were negligible, with the improvement arising purely from favorable convective patterns.^49^

To experimentally evaluate the role of the flow rate in catalytic efficiency, reactions were conducted at three different rates: 60, 120, and 240 ml min^−1^ (Fig. 4C). The highest yield of 99% was achieved at 60 ml min^−1^, which corresponds to a slower flow rate and a longer residence time. At 120 ml min^−1^, a moderate decrease in the yield to 93% was observed, while at 240 ml min^−1^, the reaction performance dropped significantly. Flow rates < 60 mL min^−1^ were not investigated, as preliminary trials showed that the relatively heavy catalyst particles did not circulate efficiently and tended to sediment rather than remain suspended. To explain these data, particle tracing simulations revealed that as the flow rate increases (60, 120, and 240 ml min^−1^), the residence time shortens dramatically: from 1.78 s to 0.48 s in backward flow, with particle retention in the illuminated region after 5 s decreasing from 21% to 6% (Fig. 4D and Fig. S12). This becomes especially critical in plasmonic catalysis, where the initial hot carrier generation and charge redistribution (fs/ps time scale) are followed by a multistep surface reaction sequence involving substrate adsorption and oxidative addition at Pd sites.^16^ While our rough estimate based on turnover frequency (SI Note S3) suggests that a full catalytic cycle might occur every ∼38 s, recent kinetic studies indicate that even the oxidative addition step alone may require >0.5 s under non-assisted conditions.^50^ This highlights a kinetic limitation in which excessively high flow rates reduce the residence time of reactants at the catalyst surface, preventing completion of the full catalytic cycle despite efficient excitation. Backward flow offers the best compromise, enhancing substrate delivery without prematurely displacing reactants from the catalytic interface. Optimal performance was achieved at 60 mL min^−1^; beyond this, the residence time falls below the minimum needed for complete plasmonic activation and reaction.

As a result of theoretical and experimental optimization of plasmon-driven Suzuki coupling, we continued our experiments under optimized conditions, namely configuration 3, 60 mL min^−1^ flow. A comparison of biphenyl formation in the batch and flow reactors under identical illumination and composition conditions demonstrates that continuous flow operation enables higher reaction rates and 3 times faster full conversion (Fig. 3B). These findings align with recent reports on reconfigurable photoflow platforms^46^ and Corcoran^51^*et al.*'s photon-equivalent-based scaling strategy,^46^ showing how tailored reactor geometry and light management can drastically enhance photoreactivity.

To exclude any side photochemical or leached-Pd homogeneous catalysis, the catalyst was removed from the reaction system after 50 min of illumination (50% BP yield). This led to an immediate cessation of biphenyl formation. The product yield remained at the same level during the next 3 hours 10 min of reaction (Fig. S13), which confirms that the catalyst must be physically present in the illuminated zone to sustain the reaction, ruling out significant Pd leaching or thermal photoeffects. While our Au@Pd catalyst is designed for heterogeneous plasmonic Suzuki coupling, a mixed pathway involving transient Pd leaching cannot be fully excluded. Such soluble Pd species, if formed, are typically short-lived and may undergo re-adsorption near the catalyst surface.^52^ Another control experiment was performed to verify that the observed coupling product originates from cross-coupling (not homocoupling). A control reaction was performed using 4-bromotoluene instead of bromobenzene for 4 hours showed similar results; the main product detected by GC-MS was 4-methylbiphenyl, not biphenyl. This confirms that the reaction proceeds *via* selective Suzuki-type C–C cross-coupling between the aryl halide and phenylboronic acid and is not a result of undesired homocoupling pathways.

### Benchmarking of catalytic parameters: AQY and TON

To evaluate the efficacy of plasmon-driven Suzuki coupling, the apparent quantum yield (AQY)^53^ and the turnover number (TON) were calculated as descriptors of catalyst activity (Table 1). The AQYs and TONs were calculated using eqn (1)–(3) and (4), respectively.^2^ In batch mode, the catalyst achieved an AQY of 2.3% for biphenyl formation, exceeding previously reported values for bimetallic catalysts irradiated with >420 nm LEDs by a factor of 1.5–23.^17–21,53^ Reported AQYs in those systems typically range from 0.1% to 9.2%, even when using higher Pd loadings or more intense light sources. Although, the 9.2% AQY in ref. 20 is higher than that in our case, the TON is much lower. At the same time, the obtained TON = 833 competes only with the value of 1527 obtained using the Au–Pd@h-CeO_2_ catalyst under Xe lamp illumination (with filter >420 nm),^19^ while the AQY in such case is relatively lower. Shifting from the batch to flow regime, the TON for plasmon-driven Suzuki coupling remained the same, indicating that the same number of Pd active sites was involved in both cases, but photon utilization was more efficient in flow mode. The AQY increased threefold (6.4%) in the flow mode compared to that in batch mode; this suggests that the improved AQY arises from the enhanced light utilization and reactant's delivery efficiency, not from an increase in catalytic capacity (Fig. 3B). Thus, the Au@PdNPs–Al_2_O_3_ sample exhibits high catalytic activity in the plasmon-assisted reaction in flow mode, giving better energy utilization with a smaller amount of the catalyst.

**Table 1:** Comparison of the AQYs and TONs of previously reported plasmonic catalytic systems

Structure and Pd, wt%	Time, h	Light source wavelength, nm	Laser power, mW cm^−2^	Yield, (amount, mmol)	Apparent quantum yield^a^, %	TON	Ref.
Pd93Au7/BiOB, 0.00035	6	400–800	300^b^	99% (0.19)	1.45	465	17
PdAu nanosheets, 0.76	12	Xe lamp	300	99% (0.25)	0.49	3.44	54
T-Pd@CN/PLA fiber, 0.64	0.67	LED lamp	360	99% (0.495)	0.1	830	18
Au–Pd@h-CeO_2_, 0.26	3	Xe lamp (filter > 420)	400	94% (0.188)	0.8	1527	19
Pd/Au NRs-*x*@rGO^c^, 0.80	0.5	LED > 420	100	65% (0.065)	9.2	16	20
AuPd/SiO_2_, 12.5	8	LED > 420	100	79%, (0.063)	0.4	240	21
**0.09**	**11**	**690**	**300**	**Yield 99% (1.6)**	**2.3**	**833**	**This work (batch)**
**0.09**	**4**	**690**	**300**	**Yield 99% (1.6)**	**6.4**	**833**	**This work (flow)**

a AQY was calculated per 1 cm^2^.

b In the absence of reported data, a laser power of 300 mW cm^−2^ was assumed.

c Iodobenzene was used instead of bromobenzene in the reaction.

### Plausible mechanistic contribution of mass transfer to flow-mode plasmon catalysis

First, to confirm the plasmonic origin of catalytic activity in our flow reactor, we evaluated the AQY at multiple excitation wavelengths. In addition to the 690 nm LED used, 590 nm and 808 nm light sources were used (Fig. S14), which fall outside the main plasmon resonance band of Au@PdNPs–Al_2_O_3_, and they produced lower AQYs (5.1% and 2.9%, respectively) compared to the 690 nm LED. In contrast, the highest AQY of 6.4% was achieved at 690 nm, which coincides with the plasmonic absorption peak of the catalyst. Although photons at 590 nm possess higher energy, the AQY at 690 nm is higher because of the excitation of the plasmonic mode hybridized with Pd–Au interfacial states, which is represented by a broad shoulder feature in the UV-vis spectrum at ∼700 nm (Fig. 1F). This observation supports the hypothesis that plasmon excitation is maximized when the illumination matches the hybridized plasmonic resonance. The utilization of a 690 nm LED source provided energy transfer *via* hot electron generation in hybridized Au–Pd modes due to the fact that different electronic behaviors favored the formation of an Ohmic contact between the two materials, which is beneficial for carriers transfer.^55,56^

**Fig. 5:**
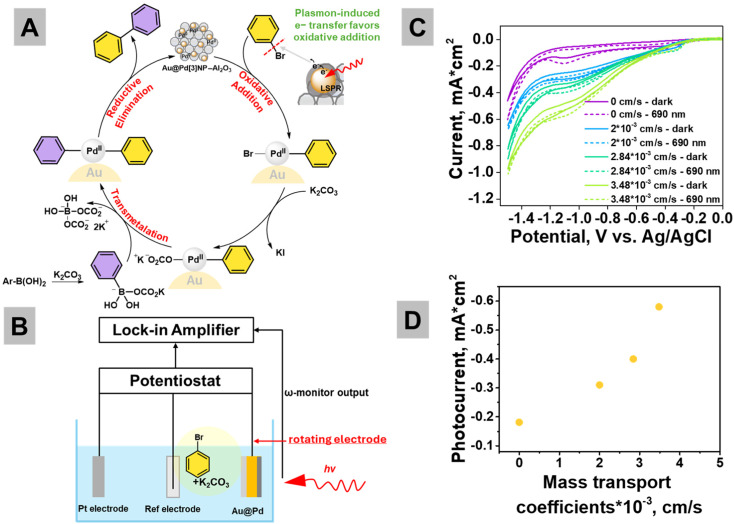
(A) Plausible catalytic cycle for plasmon-assisted Suzuki coupling where, under illumination, hot electrons generated by the Au core facilitate the oxidative addition of aryl halides at Pd sites, accelerating C–Hal bond cleavage. (B) Photoelectrochemical setup to probe light-induced charge dynamics. A three-electrode configuration with a Pt counter electrode, an Ag/Ag^+^ reference electrode, and an Au@Pd working electrode was irradiated with 690 nm light to monitor the photocurrent response. (C) CVA curves measured in the dark and under a 690 nm LED at different mass transfer coefficients (due to electrode rotation). (D) Effect of mass transfer coefficient variation on the photocurrent produced at 690 nm LED irradiation at around −1.1 V *vs.* Ag/AgCl.

Second, previous reports on the plasmon-driven Suzuki reaction revealed oxidative addition to be the rate-determining step.^57,58^ In bimetallic Au@Pd systems, Au acts as a plasmonic antenna, generating hot electrons upon light excitation. These hot electrons can transfer across the Au–Pd interface into the Pd shell, increasing its electron density and reactivity.^52,53^ The generated carriers are transferred to the aryl halide, promoting C–Hal bond cleavage^1,16^ (Fig. 5A). This activation results in the formation of a phenyl radical, which subsequently reacts with Pd(0) to form an aryl–palladium intermediate. Thus, in the Au@Pd system, plasmonic excitation facilitates both electron activation of the Pd surface and substrate activation, driving the oxidative addition step.^59,60^ This mechanism is supported by Qi Hao *et al.*, who demonstrated that plasmonic hot electrons mediate the cleavage of the C–Hal bond, a key prerequisite for the coupling reaction.^61,62^

To probe the role of mass transport limitations in plasmon-driven Suzuki coupling, we employed photocurrent measurements as an indirect indicator of hot electron transfer efficiency under varying transport conditions (Fig. 5). The aim was to probe whether the rate of reactant delivery to the catalyst surface is crucial for plasmonic chemistry. If the activation of aryl halides by hot electrons is not dictated solely by the carrier generation rates, but also by the local concentration of reactive species, then photocurrent responses should exhibit sensitivity to mass transport. By correlating the photocurrent with controlled variations in convective flow, we assess to what extent hot electron-driven reactivity is limited by substrate availability at the catalyst interface. To evaluate the hot electron transfer efficiency of the Au@Pd catalyst system under plasmonic excitation, bromobenzene was selected as the molecular probe. We study the efficiency of cleaving the C–Br bond under plasmonic conditions using the experimental setup (Fig. 5B) based on a previous report for hot electron-driven chemistry.^62^

To evaluate the influence of mass transport on plasmon-assisted C–Br cleavage, cyclic voltammetry (CVA) was conducted under LED illumination with various rotating disk speeds: 100, 200, and 300 rpm, corresponding to calculated mass transfer coefficients^63^ (*k*_m_) of 2.01 × 10^−3^, 2.84 × 10^−3^, and 3.48 × 10^−3^ cm s^−1^, respectively (Fig. 5C). These values were derived using the Levich equation, assuming diffusion-limited transport in an aqueous medium (Note S4). In the absence of rotation and illumination, a small cathodic peak is observed at around −1.1 V *vs.* Ag/AgCl (Fig. 5C and Fig. S15), corresponding to the onset of bromobenzene reduction. Upon 690 nm illumination, the photocurrent increases notably, suggesting enhanced activation of the C–Br bond at the Au@Pd surface. This enhancement may originate from plasmon-induced hot electrons injected into bromobenzene or from field-mediated excitation of the molecule or Pd^0^ species formed *via* Au → Pd electron transfer. In contrast, without illumination, the activation is significantly slower and the current remains lower.

When electrode rotation is introduced to modulate mass transport, an overall increase in current (Fig. 5C and D) is observed across the potential range of −0.25 to −1.5 V, attributed to the improved bromobenzene delivery to the electrode surface and thinning of the diffusion layer. Notably, the peak area in the −1.1 V region increases with raise of the rotation speed (Fig. 5C and Fig. S15), indicating that plasmon-driven activation could be mass-transport-limited, since the enhanced substrate availability leads to a greater extent of plasmon-activated C–Br bond cleavage. These multiple differences in transport capacity explain the enhanced delivery of bromobenzene and support the hypothesis that short-lived hot-electron-driven reaction intermediates benefit from rapid replenishment of the substrate (even the *k*_m_ of RRDE is a few orders of magnitude lower than that of the real flow reactor).

Finally, we compared the diffusion-limited flux (*J*_diff_) with the experimentally observed plasmon-catalytic flux (*J*_PC_) (Fig. S16 and 17, Table S3 and Note S5). The *J*_diff_ value was calculated using Fick's first law based on experimentally derived values for the diffusion coefficient (calculated based on the Randles–Sevcik equation^64^), concentration, and diffusion layer thickness (2.95 × 10^−9^ mol cm^−2^ s^−1^). The plasmon-catalytic flux *J*_PC_ value was determined from kinetic measurements of product formation normalized to the estimated surface area of illuminated Au@Pd nanoparticles (1.21 × 10^−9^ mol cm^−2^ s^−1^). This analysis shows that the observed catalytic flux is of the same order as the diffusion-limited value, suggesting that mass transport can indeed significantly influence the overall reaction performance. *J*_diff_ and *J*_PC_ depend strongly on assumptions such as the diffusion layer thickness and the illuminated catalytic area.^65,66^ Because both the diffusion and the illuminated area vary during operation, the obtained flux values have to be treated with a level of one magnitude of uncertainty.

In plasmonic catalysis, photons act as reactants, and the catalytic rate is ultimately governed by the interplay between molecular diffusion (*J*_diff_) and the photon delivery rate. Hot electrons are generated (in Au@Pd) within femtoseconds of photon absorption but can only drive catalysis if a reactant is present at the surface within their lifetime. Thus, the observed macroscopic *J*_PC_ reflects the overlap between fs-scale carrier generation events and reactant arrival *via* diffusion, integrated over the catalyst surface and the reaction time. Although the role of mass transport is recognized in catalysis in general, its significance has not been extensively addressed in plasmonic systems, which have traditionally focused on excited carriers dynamics. To probe this interplay, we scaled the plasmon-driven Suzuki reaction from 20 mL to 40 mL, increasing the catalyst and reactants proportionally to maintain constant concentrations. Despite a constant *J*_diff_, the biphenyl yield decreased over 4 hours (99% → 89% → 50%), pointing to a photon flux limitation. As the illuminated area and photon density did not scale accordingly, the frequency of hot carrier generation per site decreased, reducing the probability of overlap with reactants and lowering the *J*_PC_. This underscores that even with efficient ultrafast excitation, macroscopic reactivity remains limited by the joint supply of molecular and photonic reactants, emphasizing the importance of optimized reactants and light delivery in scalable plasmonic systems. To maintain high yields at larger volumes, future reactor designs should incorporate expanded illuminated surface areas (*e.g.*, modular flat-plate channels), light redistribution elements to minimize shading, and intensified mixing strategies to ensure uniform photon access across the slurry. Such approaches will be essential for translating plasmonic catalysis to scalable, high-throughput operation.

## Conclusion

This study moves plasmonic catalysis beyond its conventional boundaries by bridging plasmon catalyst design and mechanistic studies with a scalable flow concept. Through the integration of gram-scale Au@PdNPs–Al_2_O_3_ catalysts into a continuous-flow platform, we demonstrate a high-yield/AQY/TON Suzuki coupling under visible light and establish a mechanistic basis for performance enhancement due to mass transport and photon dynamic/utilization. Crucially, we show that hot electron transfer to molecular reagents, not just across metal–metal interfaces, is essential for rate acceleration in plasmonic chemistry, exemplified by the Suzuki coupling reaction. By combining CFD-guided flow reactor optimization with photoelectrochemical validation, we uncover that catalytic performance is governed by a balance between the photon residence time and substrate accessibility guided by mass transport. The identification of a critical exposure threshold (∼0.5–1.0 s) by CFD redefines how future photoreactors must be designed, not merely to illuminate, but to kinetically support plasmon-induced reactivity. For the first time, we showed that plasmon-catalytic flux and diffusion flux are within one order of magnitude, indicating that mass transport is a key limitation and that photon flux alone is insufficient to drive high catalytic turnover if reactants are not continuously delivered.

Beyond demonstrating record catalytic metrics of the AQY and the TON, this work offers a conceptual shift: light-driven reactions should be engineered as spatiotemporally resolved processes, where dynamic flow not only enhances mass transport but fundamentally could modulate the mechanism of photoactivation. To overcome the observed yield decrease with increasing reaction volume, one must scale photon delivery organization. This could include optimizing the plasmonic catalyst, light intensity, spatial coverage, residence time, and catalyst accessibility, ensuring that hot carriers generated on the fs scale are effectively coupled with reactants arriving on the ms to s scale.

## Methods

### Materials

Deionized water, methanol (puriss, p.a., absolute, ≥99.8% (GC)), ethanol (≥99.5% (GC)), chloroauric acid tetrahydrate (HAuCl_4_·4H_2_O, 99.9%), ascorbic acid (AA, 99.0%), tri-sodium citrate dihydrate (Na_3_C, 99%), aluminum oxide (Al_2_O_3_, activated, Brockmann I, standard grade, neutral, 199974, 99.5%), palladium(ii) chloride (PdCl_2_, 99%), hydrochloric acid (HCl, 36%), bromobenzene (for synthesis), phenylboronic acid (purum, ≥97.0% (HPLC)), and potassium carbonate (K_2_CO_3_, ACS reagent, ≥99.0%) were purchased from Sigma-Aldrich and used without further purification.

### Synthesis of gold nanoparticles (AuNPs)

Gold nanoparticles (AuNPs) were synthesized by the microwave-assisted method with some modification.^30^ Briefly, 1 mL of HAuCl_4_ water solution (0.0254 mmol) and 1 ml of sodium citrate solution (0.0287 mmol) were added to 18 mL of H_2_O. The resulting solution was placed in a microwave oven for 3 min at 240 W and washed with water.

### Synthesis of AuNPs–Al_2_O_3_

First, powdered Al_2_O_3_ was activated with methanol at room temperature (RT) for 30 min and dried at 60 °C. Afterwards, 18 mL of distilled water was mixed with 1 mL of HAuCl_4_ solution (0.0254 mmol), 1 mL of sodium citrate solution (0.0287 mmol) and 1 g of activated powdered Al_2_O_3_. The resulting solution was placed in a microwave oven for 3 min at 240 W. The AuNPs–Al_2_O_3_ powder was separated from the solution using a centrifuge (7830 rpm, 10 minutes), washed several times with methanol, and finally dried under vacuum.

### Synthesis of Au@Pd[1–4]NPs–Al_2_O_3_

A Pd shell was grown on AuNPs–Al_2_O_3_ according to ref. 40. Briefly, 0.2 g of AuNPs–Al_2_O_3_, 1.0 mM H_2_PdCl_4_ (0.22, 0.55, 1.34 and 2 mL) and water (9.66, 9.15, 7.96 and 7 mL), respectively, were mixed and cooled down in an ice bath. Afterwards, 10 mM ascorbic acid (0.12, 0.3, 0.7 and 1 mL) was added slowly and the solutions were left for 15 min. The Au@Pd[1–4]NPs–Al_2_O_3_ powder was separated from the solution using a centrifuge (7830 rpm, 10 minutes), washed several times with methanol, and finally dried under vacuum.

### Upscaling procedures for the synthesis of Au@Pd[3]NPs–Al_2_O_3_

3 g of AuNPs–Al_2_O_3_, 20 mL of 1.0 mM H_2_PdCl_4_, and 114.4 mL of water were mixed and cooled down in an ice bath. Afterwards, 10.5 mL of 10 mM ascorbic acid was added slowly and the solution was left for 15 min. The Au@Pd[3]NPs–Al_2_O_3_ powder was separated from the solution using a centrifuge (7830 rpm, 10 minutes), washed several times with methanol, and finally dried under vacuum.

### General procedure for the Suzuki reaction in a flask

Suzuki–Miyaura coupling was conducted in a flask (50 mL volume). In a typical reaction, bromobenzene (1.6 mmol, 1 equiv.), phenylboronic acid (3.2 mmol, 2 equiv.), K_2_CO_3_ (4.8 mmol, 3 equiv.), and Au@Pd[3]NPs–Al_2_O_3_ 0.5 g were added to 1 : 1 (v/v) H_2_O/EtOH (20 mL). The reaction mixture was stirred continuously under a 690 nm LED using a cooling fan to keep the temperature at approximately 29 °C.

### General procedure for the Suzuki reaction in continuous flow mode

The Suzuki–Miyaura cross-coupling reaction for the synthesis of biphenyl was carried out in a custom-designed photochemical flow reactor provided by Redeem Solar Technologies GmbH. This laboratory-scale reactor is constructed from stainless steel and features a rectangular flow channel equipped with arrow-shaped static mixing elements (see Fig. 3A). The flow channel is sealed with an 8 mm-thick borosilicate glass window, providing a total window area of 26.4 cm^2^ and an illuminated channel area of 20.8 cm^2^. The depth of the illuminated flow channel is 1 mm, resulting in a total illuminated reactor volume of 2.08 cm^3^. Sealing is achieved using a Viton O-ring and the reactor is mounted on a stand commercially available from the manufacturer, tilted at an angle of 60° along the inlet–outlet axis. A peristaltic pump is used to circulate the slurry from the reactor to a stirred glass vial and back. The slurry is introduced into the reactor through the bottom inlet and flows upward toward the top outlet. The reactor is irradiated using the same light source as employed for batch reactor screening experiments and is cooled using a fan to maintain the reactor temperature within the range of 25–28 °C. The arrow reactor was investigated under two distinct configurations: (1) arrows aligned in the direction of flow (upward orientation) and (2) arrows oriented counter to the flow direction (downward orientation). These two configurations were also repeated with the slurry introduced from the top of the reactor, yielding four experimental conditions in total.

In a typical experiment, bromobenzene (1.6 mmol, 1 equiv.), phenylboronic acid (3.2 mmol, 2 equiv.), and potassium carbonate (K_2_CO_3_, 4.8 mmol, 3 equiv.) were dispersed in a 1 : 1 (v/v) H_2_O/EtOH solvent mixture (20 mL total volume). The heterogeneous catalyst, Au@Pd[3]NPs supported on Al_2_O_3_ (0.5 g), was added to the reaction mixture, which was subsequently pumped through the reactor using a peristaltic pump (flow rate of 60 mL min^−1^).

The reactor was continuously irradiated with a 690 nm LED light source (300 mW cm^−2^) at 1 cm distance from the reaction mixture for 4 hours while being actively cooled using an axial fan (D1K1 AC 220 V PC fan) to maintain thermal stability.

Quantitative analysis of biphenyl was performed by gas chromatography-mass spectrometry (GC-MS). The sampling (0.5 mL) was performed over a certain period of time and the aliquot was extracted with hexane (1 mL). A sample of the reaction mixture was taken and analyzed *via* GC-MS using a Shimadzu Nexis 2030 gas chromatograph equipped with an SH-5MS capillary column (30 m × 0.25 mm i.d., 0.25 μm film thickness). Helium was used as the carrier gas at a pressure of 119.0 kPa and a linear velocity of 49.7 cm s^−1^. The heat starts at 40 °C (for 2 min) following with temperature increase till 280 °C (20 °C min^−1^ rate) and finish with 280 (8 min). The injection volume was 1 μL, with a split ratio of 40 : 1. The inlet temperature was 280 °C and the *m*/*z* range was 10–500. GC calibration was performed before each series of experiments to ensure quantitative accuracy.

### Control experiment in the dark

Bromobenzene (1.6 mmol, 1 equiv.), phenylboronic acid (3.2 mmol, 2 equiv.), and potassium carbonate (K_2_CO_3_, 4.8 mmol, 3 equiv.) were dispersed in a 1 : 1 (v/v) H_2_O/EtOH solvent mixture (20 mL total volume). The heterogeneous catalyst, Au@Pd[3]NPs–Al_2_O_3_ (0.5 g), was added to the reaction mixture, which was subsequently pumped through the reactor using a peristaltic pump (flow rate of 60 mL min^−1^) for 4 hours.

### Control experiment without the catalyst

Bromobenzene (1.6 mmol, 1 equiv.), phenylboronic acid (3.2 mmol, 2 equiv.), and potassium carbonate (K_2_CO_3_, 4.8 mmol, 3 equiv.) were dispersed in a 1 : 1 (v/v) H_2_O/EtOH solvent mixture (20 mL total volume), which was subsequently pumped through the reactor using a peristaltic pump (flow rate of 60 mL min^−1^) for 4 hours.

### Control experiment with bromotoluene

Suzuki–Miyaura coupling was conducted in a flask (50 ml volume). In a typical reaction, bromotoluene (1.6 mmol, 1 equiv.), phenylboronic acid (3.2 mmol, 2 equiv.), K_2_CO_3_ (4.8 mmol, 3 equiv.), and Au@Pd[3]NPs–Al_2_O_3_ 0.5 g were added to 1 : 1 (v/v) H_2_O/EtOH (20 mL). The reaction mixture was stirred continuously under a 690 nm LED using a cooling fan to keep the temperature at approximately 29 °C.

### Photocamera measurement

The surface temperature changes during the experiment were monitored using an FLIR E5 infrared camera.

### Procedure to calculate the AQY

The apparent quantum yield (AQY) was calculated according to the following equation:1
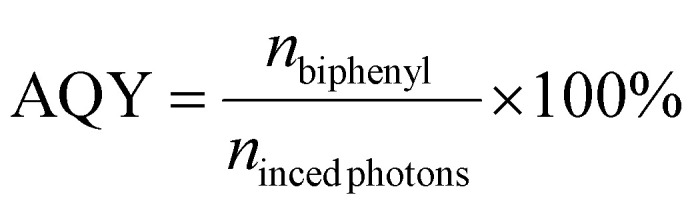
where *n*_biphenyl_ is the number of moles of formed biphenyl and *n*_inced photons_ is the number of moles of incident photons.

The number of incident photons (in moles) was calculated from the measured power and the averaged photon energy:2
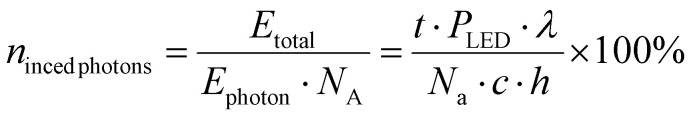
where *λ* is the wavelength of light (m), *P* is the power of the light source (W), *t* is the reaction time under illumination (s), *h* is Planck's constant (6.63 × 10^−34^ J s^−1^), *c* is the speed of light (2.998 × 10^8^ m s^−1^), and *N*_A_ is Avogadro's number (6.022 × 10^23^ mol^−1^).

In the case of the Xe lamp, the averaged photon energy was calculated as a weighted sum of the photon energies at each wavelength (*E* = *hc*/*λ*), using the normalized spectral irradiance (Fig. S18) to represent the relative contribution of each wavelength to the total irradiance.^36^

For example, *E*_photon_, corresponding to the irradiance range of 400–800 nm, was calculated as:3
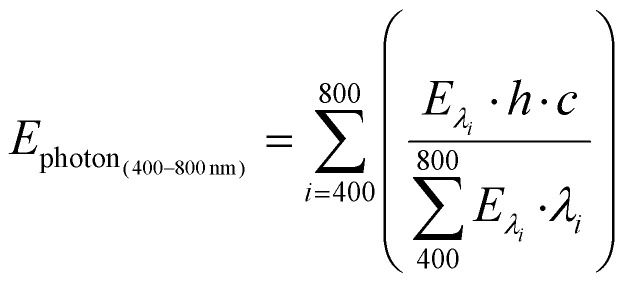
where *E*_*λ*_i__ is the spectral irradiance for each wavelength (W m^−2^ nm^−1^).

The main aim was to compare the photocatalytic performance with the literature data; the AQY was determined using a standard irradiated area of 1 cm^2^.

### Calculation of the turnover number (TON)

The TONs were calculated as a ratio, *n*_product_/*n*_cat_, where *n*_product_ is the number of the obtained product (biphenyl) and *n*_cat_ is the number of moles of the catalytic centers (Pd):

4 TON = *n*_product_/*n*_cat_

#### Transmission electron microscopy (TEM)

Transmission electron microscopy (TEM) photos were obtained using a FEI/ThermoFisher (TFS) Tecnai G2 20 (S)TEM equipped with an X-FEG electron source and operated at a 200 kV acceleration voltage. The STEM-EDX measurements were performed using an EDAX Appollo XLT windowless 30 mm^2^ SDD detector with an energy resolution of 132.9 eV.

#### UV-vis absorption spectroscopy

UV-vis absorption spectra were obtained using an HR2000 (Ocean Optics) spectrometer in the 400–1000 nm wavelength range with an AvaLight-DHS light source (Avantes).

#### X-ray diffraction data

X-ray diffraction data were collected using an XRD-diffractometer PAN analytical X'Pert PRO with Cu Kα radiation (1.5406 Å).

#### X-ray photon spectroscopy (XPS)

X-ray photon spectroscopy (XPS) spectra were obtained using a Thermo Fisher Scientific XPS NEXSA spectrometer equipped with an Al K Alpha X-ray monochromatic emitter with an energy of 1486.6 eV. Survey spectra were recorded using a pass energy of 200 eV with an energy resolution of 1 eV. The spectra were calibrated against the C1s peak set at 284.8 eV. High-resolution spectra (O1s, Al2p, Pd3d, and Au4f) were obtained using a pass energy of 50 eV and a resolution of 0.1 eV. The concentrations of elements were calculated in at% using the sensitivity factors provided by the manufacturer.

#### Scanning electron microscopy

Scanning electron micrographs were obtained using a Sigma HD-VP (Zeiss, Germany) with an Everhart Thornley SE-electron detector. Powders was fixed with carbon tape to the sample holder and analyzed without further pretreatment. Micrographs were obtained at 2–15 kV and a 3.5–8.9 mm working distance.

#### Atomic adsorption spectroscopy (AAS)

Atomic adsorption spectroscopy (AAS) spectra were obtained using an AGILENT 280 FS AA spectrometer (Agilent Technologies Australia).

### CFD simulations

Finite element method (FEM) simulations were performed using the CFD module of Comsol running on a workstation PC. Laminar flow was assumed as the corresponding Reynolds numbers of the structures (∼20) were small enough to only consider turbulent flow using a *k* − *ε* approximation. Afterwards, particle tracing was performed using the obtained flow profiles for forward and backward flows, therefore ignoring the back action of the particles on the fluid. The residence times were computed by taking the average of the integral of the time steps from the entry point to the exit at the outlet. Depending on the flow configuration, 77% to 94% (forward @100rpm and backward @300rpm respectively) of the particles exited the reactor by the end of the simulation time. This is a measure of how numerically sensible the obtained residence times are. For computational reasons, particles that had collided with a wall were not further acknowledged in the process and not considered for the average. This is assumed to be a valid approximation as the residence times with and without wall collision could be shown to be very similar.

### Electrochemical measurements

All electrochemical experiments were performed in a standard three-electrode cell. Ag/AgCl (3 M KCl) was used as the reference electrode, a platinum spring served as the counter electrode, and the working electrode was made from glassy carbon. Au@Pd–Al_2_O_3_ particles were applied to the working electrode as follows: a suspension of particles in methanol (300 µl) was mixed with 5 µl of a 5% solution of Nafion, after which the resulting solution was subjected to ultrasonic treatment for 5 minutes. Then, 10 µl of the prepared suspension was applied dropwise to the glassy carbon electrode and dried at 50 °C for 20 minutes. The electrolyte was a water–ethanol solution containing 0.1 M K_2_CO_3_ and bromobenzene at a concentration of 0.18 M. During the experiment, the sample was illuminated with an LED (690 nm, 300 mW cm^−2^). All electrochemical studies were performed using a rotating-ring disk electrode (RRDE) rotary system (100, 200, and 300 rpm) manufactured by IVIUM Technologies.

### Computational methodology

All optical simulations were performed using the TERMS (*T*-matrix for Electromagnetic Radiation with Multiple Scatterers) framework using the superposition *T*-matrix specifically designed for nanoplasmonic systems. The TERMS platform solves Maxwell's equations in the frequency domain using a surface integral formulation, which enables accurate modelling of complex core–shell geometries and near-field interactions between closely spaced nanostructures. Core–shell dimer systems were modelled as spherical Au@Pd nanoparticles consisting of a gold core (20 nm radius) surrounded by a 3 nm palladium shell. The interparticle edge-to-edge spacing was varied between 3 nm and 5 nm by adjusting the center-to-center distance of the two spheres. The dielectric environment was modelled as homogeneous with a refractive index of 1.7689, corresponding to a typical aqueous or alcohol-based solvent. Far-field scattering and absorption cross-sections were computed by enabling the *cross_sections* output option in TERMS, using *ModeAndScheme 2 2* and a multipole cutoff of 20. For near-field analysis, the electric field intensity (|*E*|^2^) and its derived quantities were mapped over a 2D plane (*z* = 0) using the MapQuantity 2 E directive and a spatial resolution of 600 × 600 points in a 140 nm × 140 nm region. Material dispersion was included *via* dielectric function files based on experimentally measured optical constants. The Raman enhancement factor was computed as |*E*|^4^ from the simulated local electric field intensities, consistent with the electromagnetic enhancement mechanism in surface-enhanced Raman scattering (SERS). Hot carrier generation was estimated using a simplified proxy, Im(*ε*) × |*E*|^2^, where the imaginary part of the complex dielectric function represents material losses contributing to non-radiative plasmon decay. The spatial distribution of this quantity was used to evaluate the localization and efficiency of hot carrier excitation within the Pd shell. All visualizations and post-processing analyses, including extraction of enhancement maps and hot carrier distributions, were carried out in **R** using custom scripts built around the *rhdf5*, *ggplot2*, and *viridis* packages.

## Author contributions

M. E. and D. V.: investigation, data curation, visualization, writing – original draft, and validation; E. M.: investigation, data curation, and visualization; O. G.: methodology; M. Y. S. I. and D. M.: resources and writing – review & editing; T. F. and C. K.: formal analysis, visualization, and investigation; J. H. M. and M. M. M.: formal analysis, investigation, and writing – original draft; M. V.: resources; O. L.: supervision, resources, and writing – review & editing; O. G.: conceptualization, supervision, and writing – original draft.

## Conflicts of interest

There are no conflicts to declare.

## Note added after first publication

This article replaces the version published on 12 December 2025, which did not include the emerging investigator's biography.

## Supplementary Material

NR-018-D5NR03832D-s001

## Data Availability

The data supporting this article have been included as part of the supplementary information (SI). Supplementary information is available, including detailed experimental methods, extended materials characterization (TEM, XPS, UV–Vis, GC), kinetic analyses, control experiments, CFD simulations, electrochemical measurements, and full calculations of mass transport, turnover frequencies, and catalytic fluxes. See DOI: https://doi.org/10.1039/d5nr03832d.
