# Supplementary material for: From batch to flow plasmon catalysis: revealing mass transport limits in Au@Pd nanocatalysts for Suzuki coupling
Source: Nanoscale. 2025 Dec 12;18(10):5357–70. doi: 10.1039/d5nr03832d (PMC12856979; doi:10.1039/d5nr03832d)
Supplement: NR-018-D5NR03832D-s001 [file NR-018-D5NR03832D-s001.pdf]

Supplementary Information (SI) for Nanoscale.
This journal is © The Royal Society of Chemistry 2025

Supplementary Information

From Batch to Flow Plasmon Catalysis: Revealing Mass Transport Limits in Au@Pd Nanocatalysts for Suzuki Coupling

Mariia Erzina[a†], Daria Votkina[b†], Elena Miliutina[a], Oleg Gorin[a], Malek Y. S. Ibrahim[c], David M. Köpfler[c], Tobias Friedl[d], Christian Koller[d,e], Junais Habeeb Mokkath[f], Mufasila Mumthaz Muhammed[g], Markus Valtiner[h], Oleksiy Lyutakov[a], Olga Guselnikova[b,h*]

[a] Department of Solid-State Engineering, University of Chemistry and Technology, Technicka 5, Prague 166 28, Czech Republic

[b] Research School of Chemistry and Applied Biomedical Sciences, Tomsk Polytechnic University, Lenina Avn. 30, Tomsk, 634050, Russian Federation

[c] Redeem Solar Technologies GmbH, Sandgasse 36/IV, 8010 Graz, Austria

[d] University of Applied Sciences Wiener Neustadt, Johannes Gutenberg-Straße 3, 2700 Wiener Neustadt, Austria

[e] Fotec - Forschungs- und Technologietransfer GmbH, Wiener Neustadt, Viktor Kaplan-Straße 22700 Wiener Neustadt Austria

[f] College of Integrative Studies, Abdullah Al Salem University, Khaldiya campus, Kuwait

[g] College of Engineering, International University of Science and Technology in Kuwait, Ardiya, Kuwait

[h] Vienna University of Technology, Institute of Applied Physics, Karlsplatz 13, 1040 Vienna, Austria

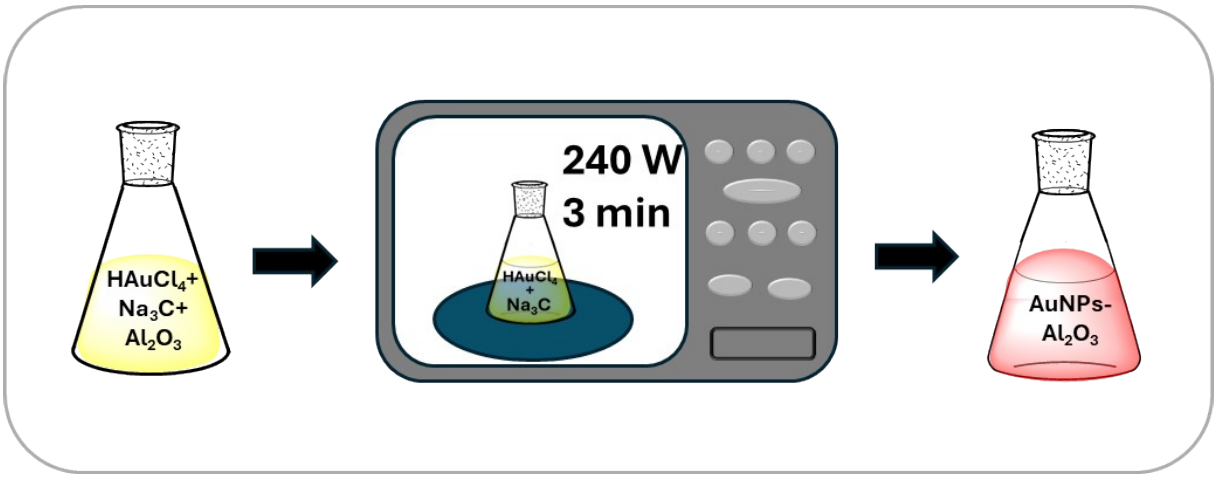

Figure S1. Simplified scheme of MW synthesis of AuNPs-Al_2O_3

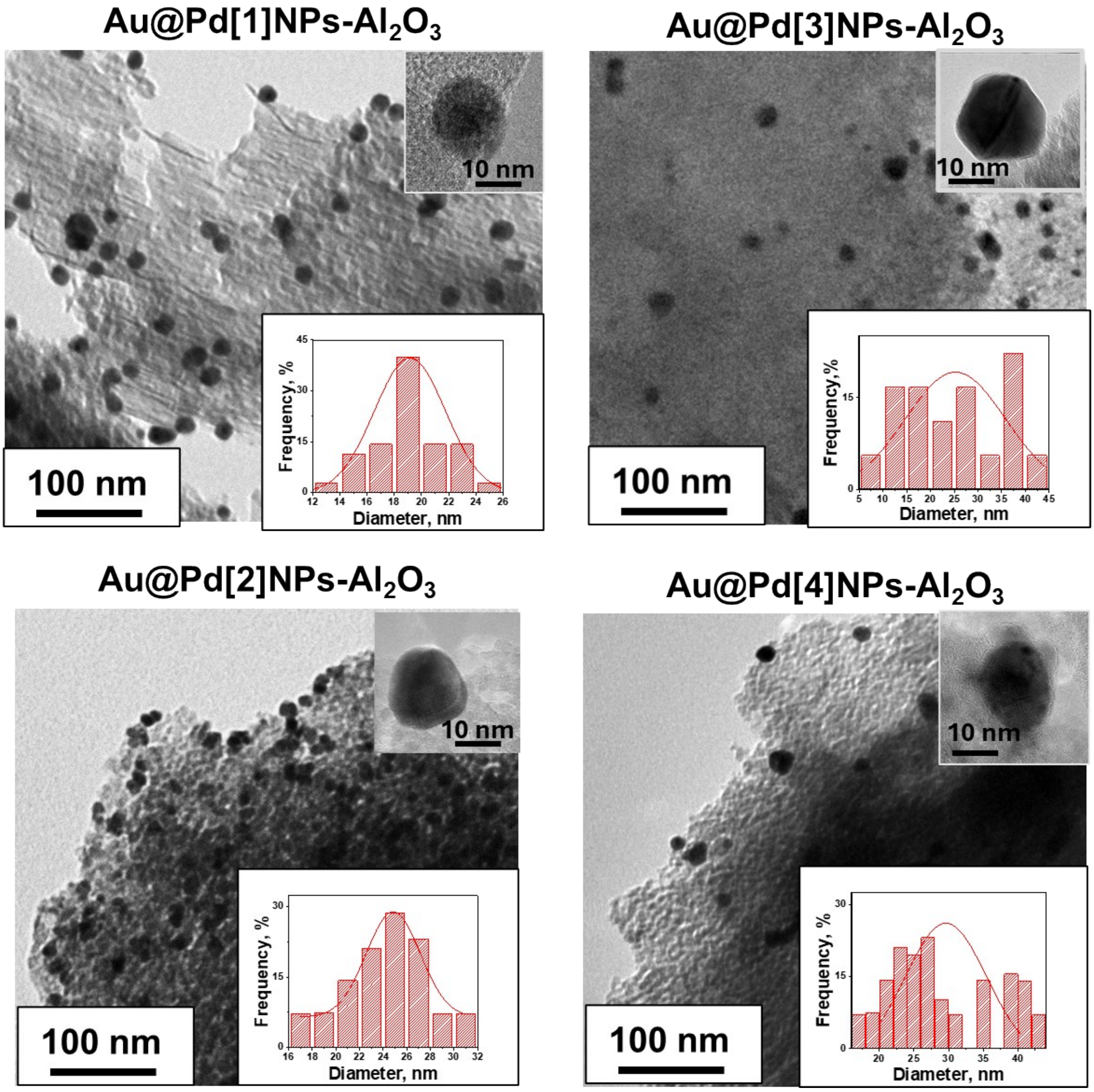

Figure S2. TEM images of thickness of Au@Pd[1-4]NPs-Al_2O_3: TEM micrographs with increasing Pd shell thickness. Inserts show higher-magnification images of individual of Au@Pd[1-4]NPs. Size distribution histograms (bottom right of each panel) correspond to the

respective TEM images, indicating average diameters and polydispersity of the nanoparticle populations.

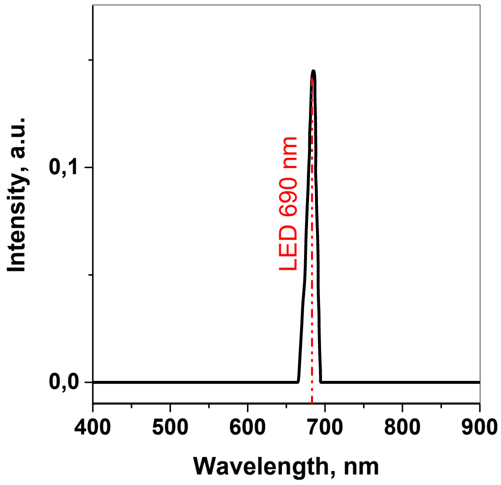

Figure S3. Irradiance profile of LED with 690 nm wavelength

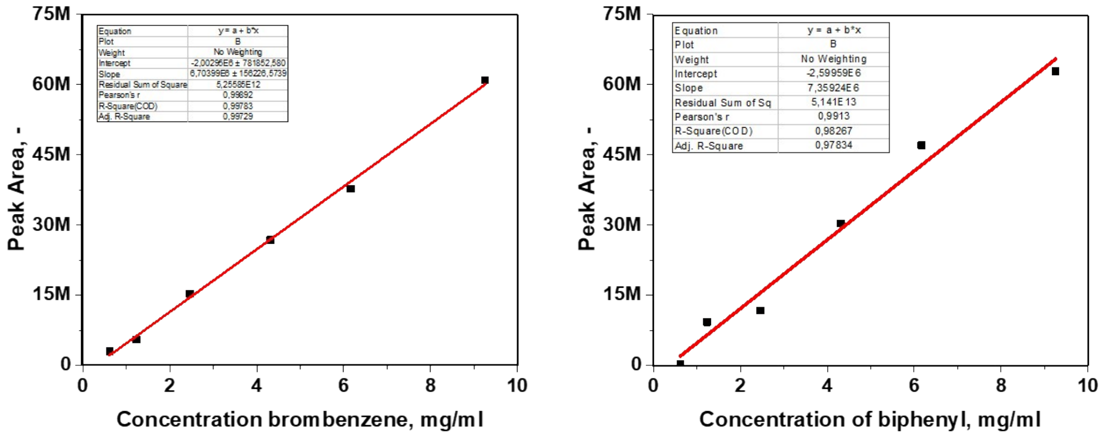

Figure S4. Calibration plot for bromobenzene and biphenyl showing a linear relationship between peak area and concentration. Red lines indicate the linear fit.

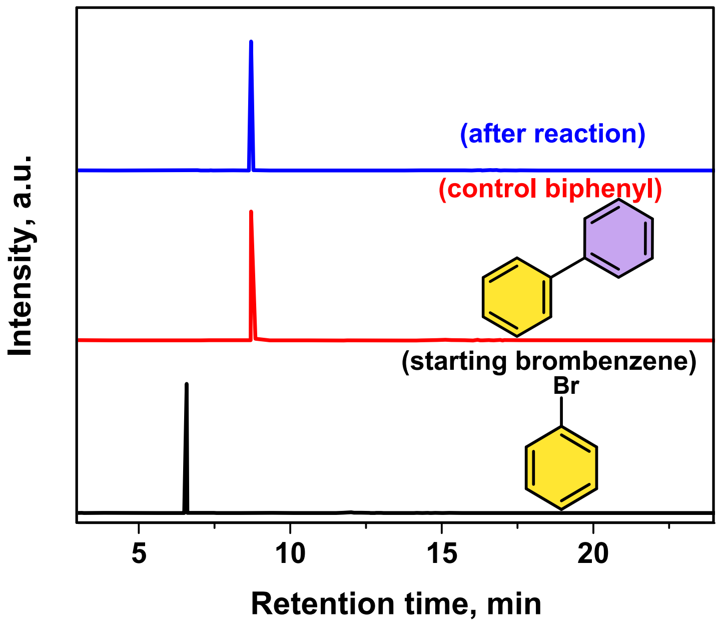

Figure S5. Representative GC chromatogram of the reaction mixture showing distinct peaks corresponding to the product (biphenyl) and starting bromobenzene. Retention times and peak identities were confirmed by comparison with standards.

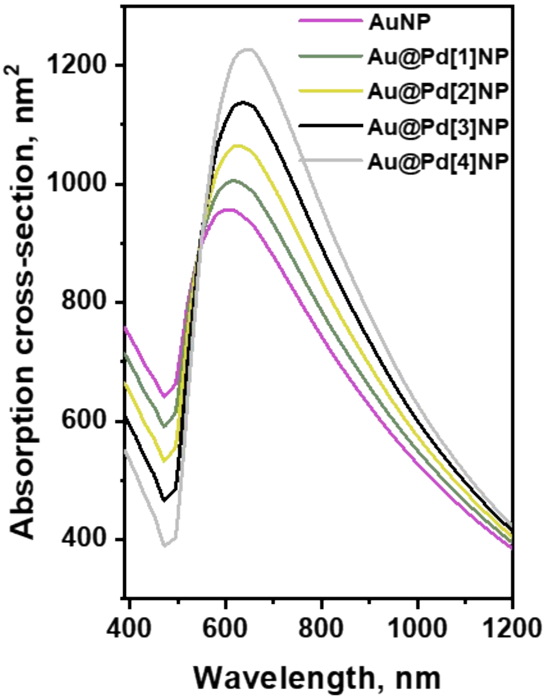

Figure S6. Simulated absorption cross-sections of Au@PdNPs with increasing Pd shell thicknesses. The spectral shift and broadening are consistent with increasing plasmon damping and interband transitions in Pd.

Table S1. Results of atomic absorption spectroscopy measurements

Sample	Ratio of Pd to Au
AuNPs-Al_2O_3	-

Au@Pd[1]NPs-Al_2O_3	-
Au@Pd[2]NPs-Al_2O_3	1:7
Au@Pd[3]NPs-Al_2O_3	1:3
Au@Pd[4]NPs-Al_2O_3	1:2

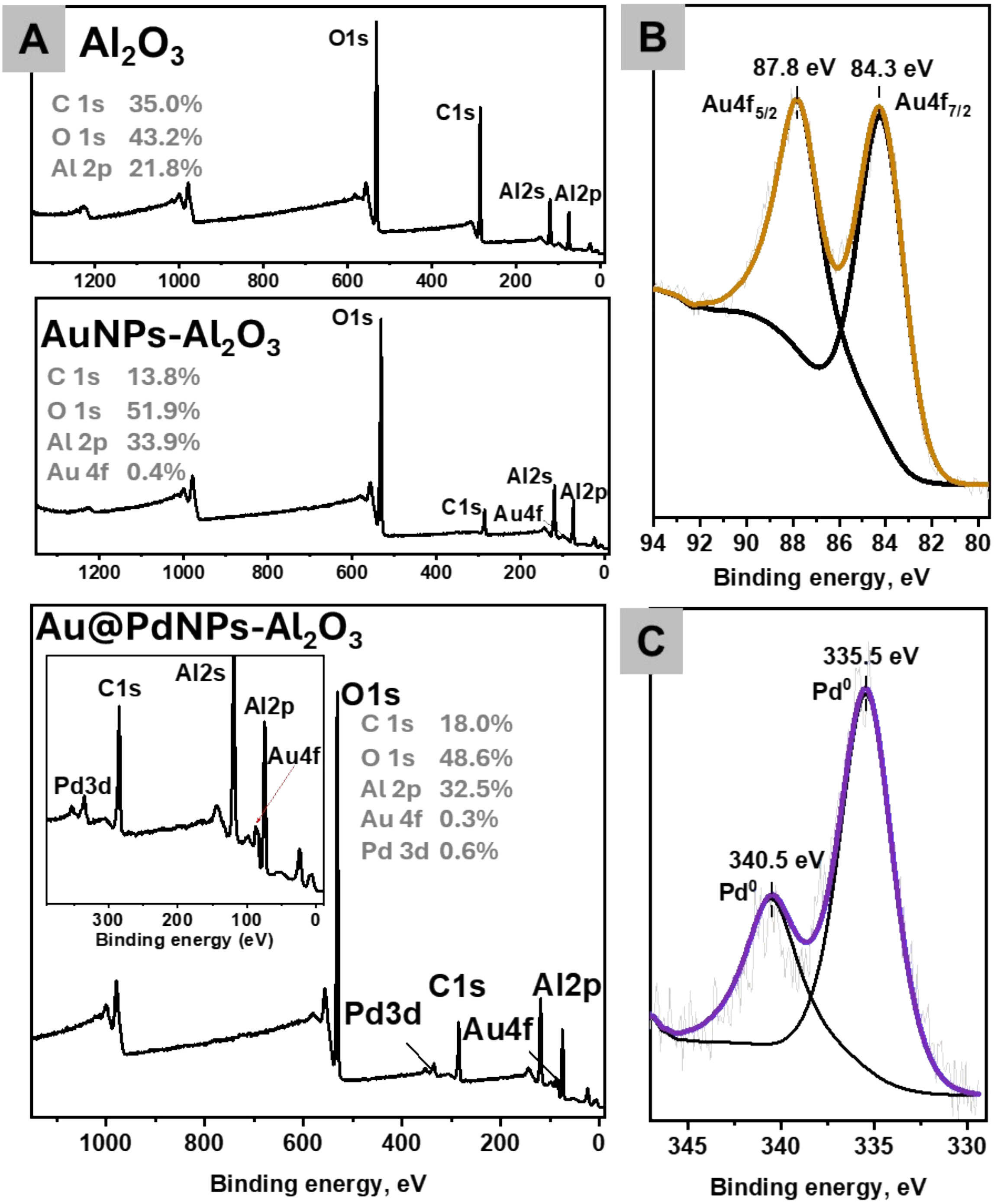

Figure S7. Characterization of Au@PdNPs-Al_2O_3 surface by XPS: A – Survey spectra of Al_2O_3, AuNPs- Al_2O_3 and Au@PdNPs-Al_2O_3 with their element composition (in %); B – High resolution of Au 4f region; C – High resolution of Pd 3d region.

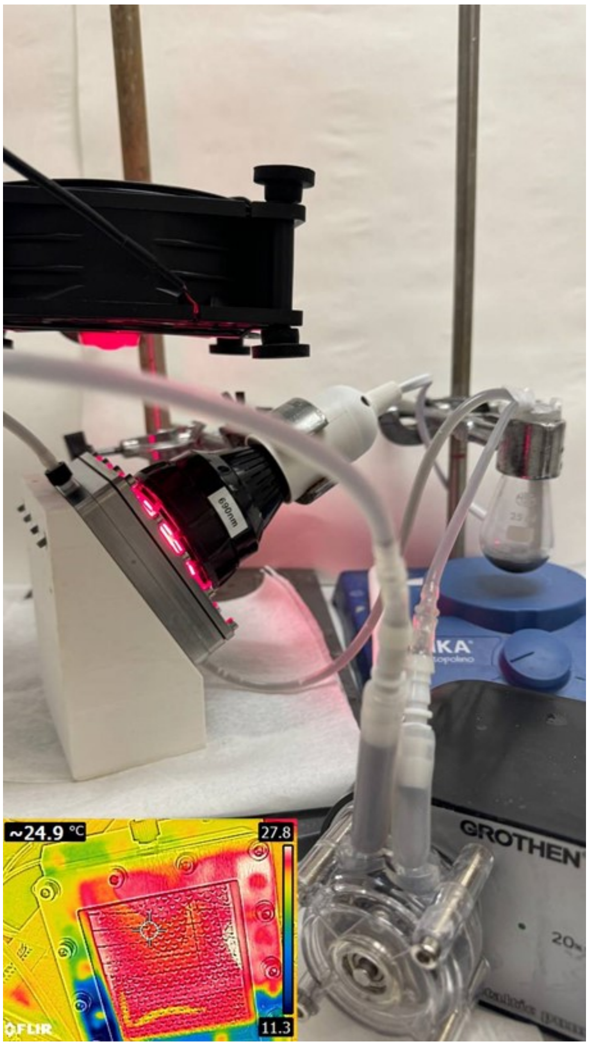

Figure S8. Photo of experimental set-up with inserted image provided by thermocamera

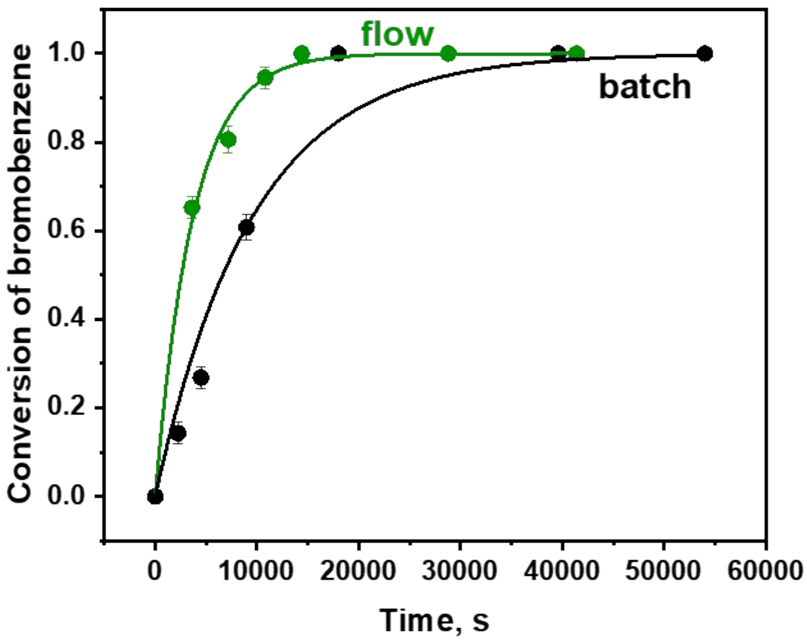

Figure S9. Kinetic curves of bromobenzene consumption represented as conversion fitted with exponential function that correspond to pseudo-first order reaction

Suppl. Note 1 – Rate constant calculation

To compare the rates of batch and flow reactor reactions, we plotted conversion of bromobenzene vs. time to estimate the appropriate constants assuming oxidative addition []. Indeed, measuring of rate constants of cross-coupling reaction is really sophisticated task requiring studying of each catalytic cycle step; thus, we focused on oxidative addition step only that could be monitored *via* bromobenzene consumption. As halogenide was in excess to palladium, we consider the reaction as pseudo-first order process. So, we applied exponential function to get rate constants 1.05×10^{-4} and 2.7×10^{-4} s^{-1} for batch and flow reactor, respectively.

Then, we normalized rate constants to illuminated area, providing appropriate values: $6.82\times10^{-5} s^{-1}/cm^2$ for batch (illuminated area 1.5 cm^2) and 1.29×10^{-5} s^{-1}/cm^2 (illuminated area 20.8 cm^2) for flow.

Suppl. Note 2 – CFD model

The results in this paper stem from CFD simulations done in 3D that used a geometry as depicted in Figure S10. Here also the skewness of the mesh is depicted in order to indicate that no numerically challenging elements were present. The basic model parameters are shown in Table S2. The simulations were done in COMSOL Multiphysics 6.2 using the Turbulent Flow, k-ε and Particle Tracing for Fluid Flow interfaces. First the Flow model was established and in a later step 40000 particles were added, so no back action of the particles on the fluid were assumed.

The amount of particles input has no correlation to the actual number of particles in the real system, but simply serve to allow averaging and such over a large basis of data. Simulating these particles as having no impact on the flow is assumed valid as the volume ratio is only ~1% in comparison to the fluid. The particle diameter given was used to calculated force coefficients for drag, buoyancy and gravity, not for the real volume used up inside the fluid.

Table S2. Model parameters used for fluid flow and particle tracing respectively.

**Fluid Flow**	Fluid density, ρ_f	Dynamic viscosity, μ	Inlet condition (vol flowrate)
	930 kg/m^3	1.9 [mPa · s]	1000|2000|3000[mm^3/s](=100|200|300[rpm])
**Particle Tracing**	Particle density, ρ_p	Particle diameter, d	Inlet condition (on inlet boundary @ simulation time 0s)
	4000 kg/m^3	22 [um]	40 000 numerical particles, initial velocity equal to flow

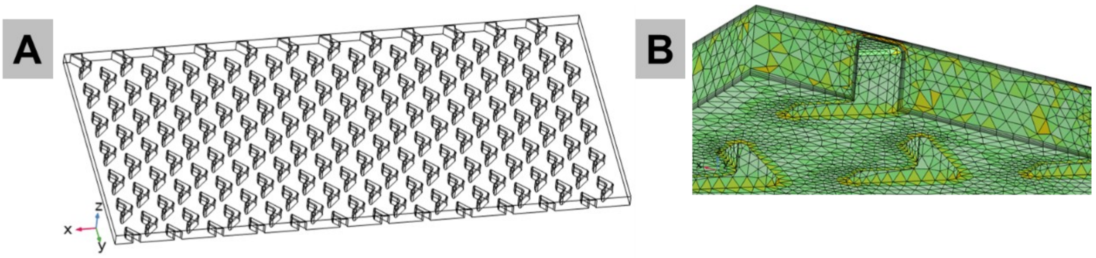

Figure S10. A - 3D-Geometry used for CFD for Redeem/TU. Symmetry on outer face in y-direction. B - Free Tetrahedral mesh and boundary layer mesh for used geometry, colored by skewness.

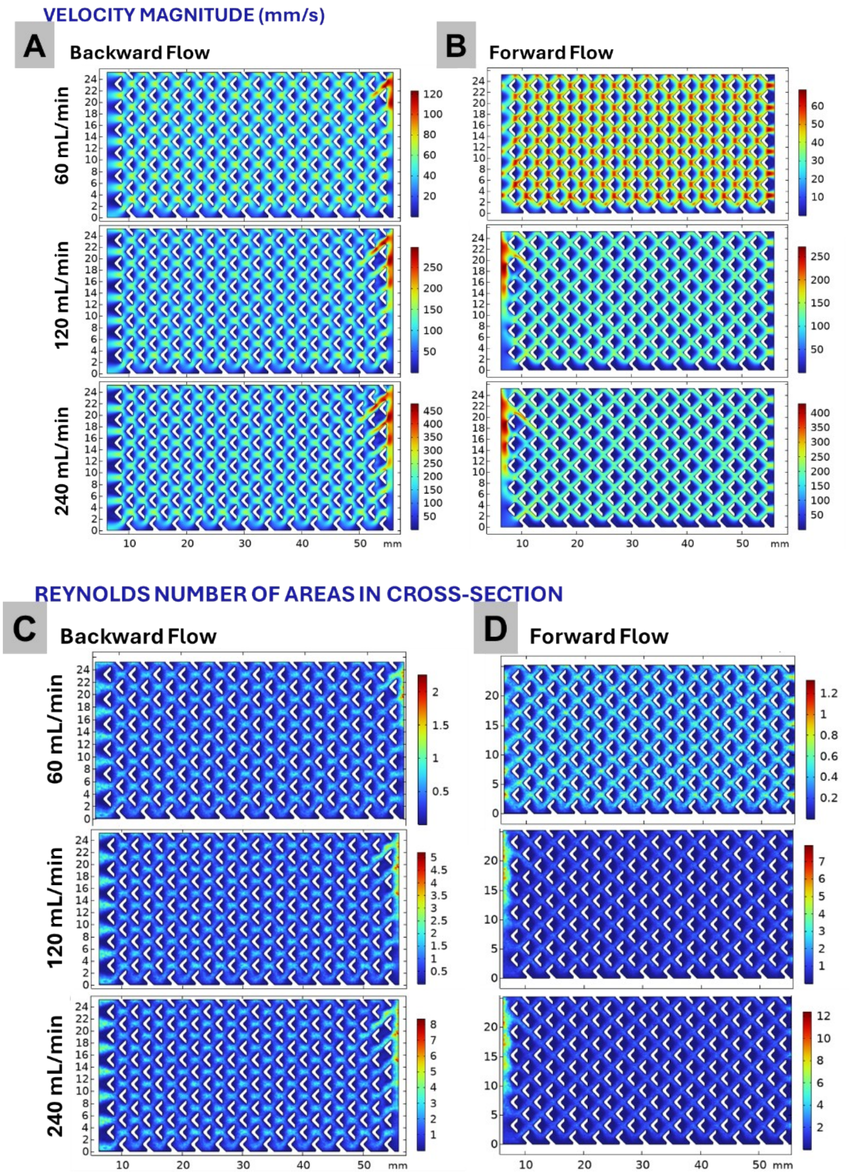

Figure S11. Flow (A and B) and Re numbers (C and D) for all flow rates and directions.

Figure S11 (A and B) illuminate the obtained flow for given flow rates within the structures for backward and forward flow as described in the main text. The inflow condition for all models was derived from the earlier simulation of a similar reactor as described in ref.[1]. Based on these profiles the local Reynold numbers could be calculated indicating the validity of the low turbulence approximation.

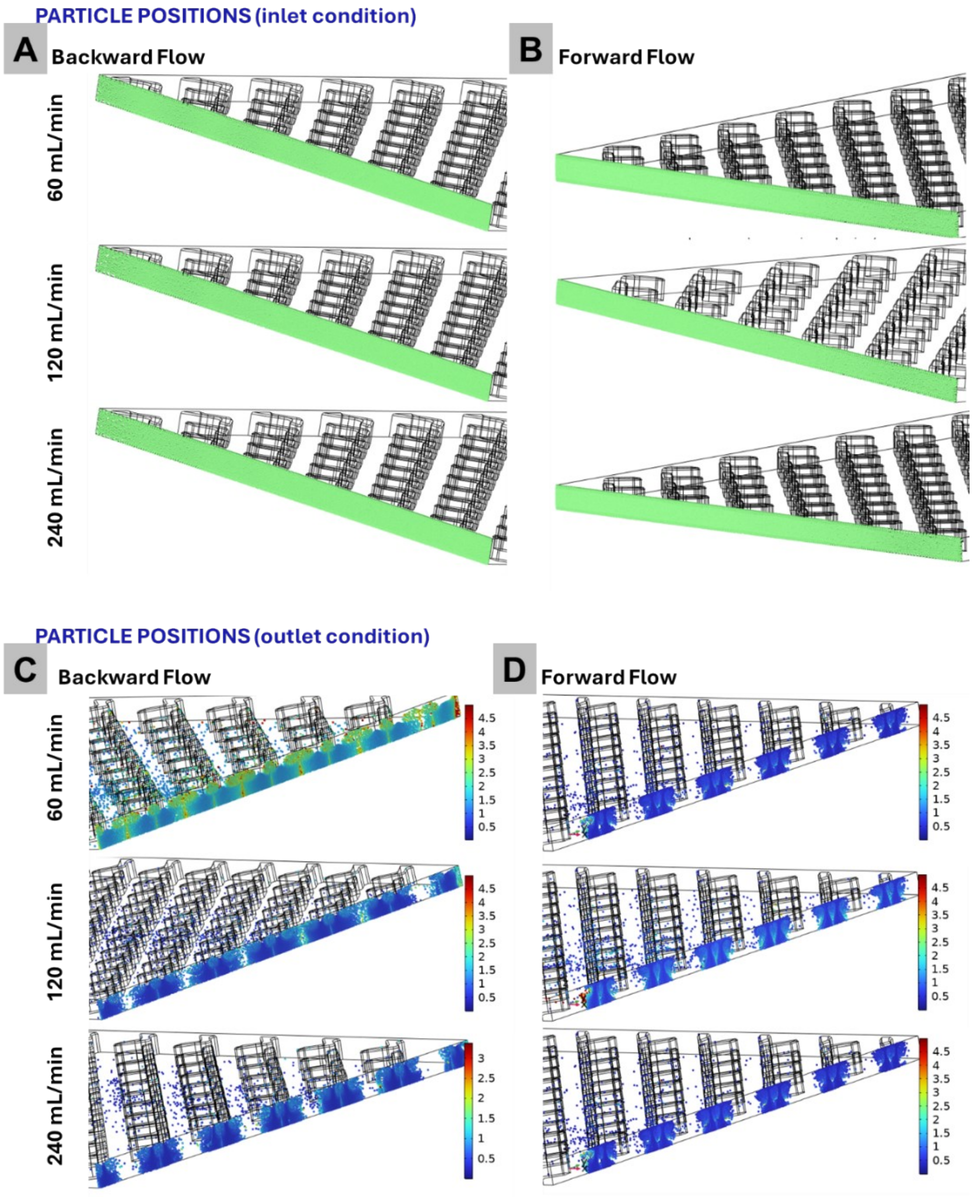

Figure S12. Particle positions at inlet (A and B) and outlet (C and D) boundary for all flow rates and directions. The C and D figures show the particle positions for their respective models at time 5s.

In Figure S12 the results of the particle tracing is shown. The particles were assumed to be evenly spread when entering the reactor (S12 A, B) The colour scale (S12 C& D) indicates residence time of each particle in seconds for both flow directions. As stated in the main text particles that interacted with boundaries where excluded form the statistic due to numerical reasons

Table S2. Parameters of flow reactor for the plasmon-driven Suzuki coupling

Parameter	Forward flow	Backward flow
Re_{avg}	0.83	0.94
Re_{max}	12.9	9.89
Flow regime	laminar	laminar
BP yield, %	~70	~90

Suppl. Note 3 – TOF calculations

**The yield of biphenyl, mol**	**1.6×10^{-3}**
**Time of the reaction, sec**	**14 400 (4 h)**
**The amount of palladium, mol**	**n (Pd) = 4.24×10^{-6}**<br>m ($Au@Pd-Al_2O_3$) = 0.5g<br>wt.% (Pd) = 0.09<br>m (Pd) = 0.5 g x 0.0009 = 0.00045 g

$$TOF = \frac{mol\ of\ product}{mol\ of\ catalyst \cdot time\ in\ seconds} = \frac{1.6 \cdot 10^{-3}}{4.24 \cdot 10^{-6} \cdot 14400} = 0.026\ s^{-1} \quad \text{(eqn. S1)}$$

Catalytic Cycle Time:

Time per cycle = 1 / TOF = 1 / 0.026 ~ 38.2 seconds

$$Time\ per\ cycle\ = \frac{1}{TOF} = \frac{1}{0.026} = 38.2\ \text{sec} \quad \text{(eqn. S2)}$$

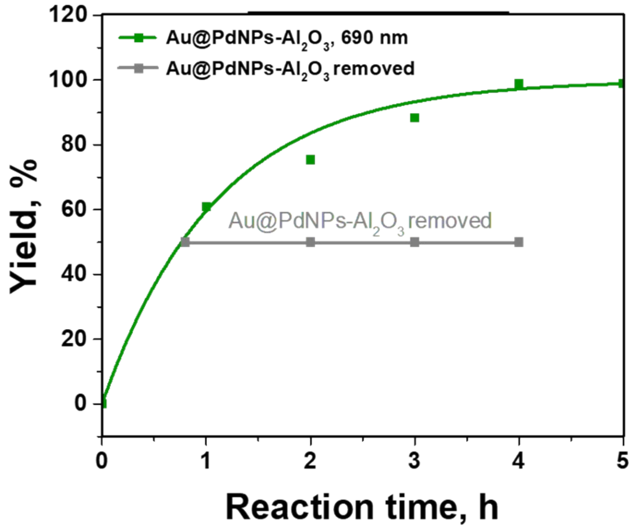

Figure S13. Results of control leaching experiment, where $Au@PdNPs-Al_2O_3$ were removed and reaction was proceeded

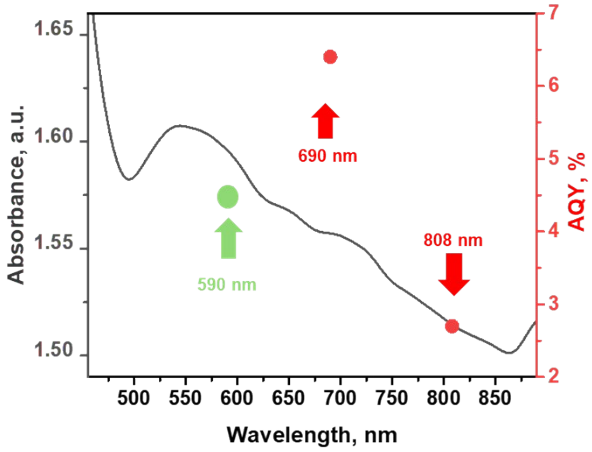

Figure S14. UV–Vis absorption spectrum of Au@PdNPs-Al_2O_3 and the apparent quantum yield (AQY) as a function of the irradiation wavelength.

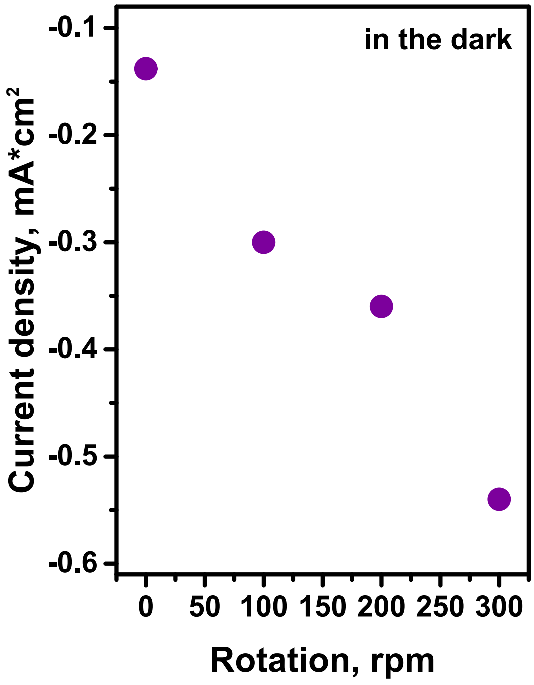

Figure S15. Dependence of current density on rotation speed measured in the dark.

Suppl. Note 4 –Estimation of Mass Transport Coefficient k_m

RRDE electrochemical system (3 mm diameter disk electrode was used):

Mass transport coefficients k_m were estimated using a standard Levich-derived expression[2] for rotating electrode:

$$k_m = 0.62D^{2/3}v^{-1/6}w^{1/2} \qquad \text{(eqn. S3)}$$

Where D=1×10^{-5} cm^2/s (diffusion coefficinet for water)

V=0.01 cm^2 (kinetic viscosity of water)

$$w = \frac{2\pi \times rpm}{60} - angular\ veolcity\ in\ rad/s \qquad \text{(eqn. S4)}$$

Resulting values are 100 rpm → k_m=2.01×10^{-3} cm/s; 200 rpm → k_m=2.84×10^{-3} cm/s; 300 rpm → k_m=3.48×10^{-3} cm/s

Flow reactor (dispersed catalyst was used, 60 mL/min)

The flow reactor consisted of a 5 cm (width) × 10 cm (length) × 1 cm (depth) channel with a suspension of 20 μm Au@Pd nanoparticles. For mass transfer to spherical particles under laminar flow, the Sherwood number correlation was used:

$$Sh = 2 + 0.6Re^{1/2}Sc^{1/3}, k_m = \frac{Sh * D}{d_p} \quad \text{(eqn. S5)}$$

Where

d_p-Au@Pd-Al2O3 particle diameter

D=1×10^{-5} cm^2/s (diffusion coefficinet for water

Re ≈0.04 Sc≈ 1000. Sh≈ 3.2

This yielded $k_m =$ k_m=2.84×10^{-3}

Suppl. Note 5. Estimation of diffusion-limited flux and comparison with observed catalytic rate

To assess the role of mass transport in the observed plasmon-assisted Suzuki coupling reaction, we calculated the theoretical diffusion-limited flux (J_{diff})[3] and compared it to the experimentally determined catalytic flux for plasmon catalysis (J_{PC}).

Diffusion-limited flux (Fick's first law)

Fick's first law is expressed as:

$J_{diff} = (D \times C_0) / \delta$

Where:

D - diffusion coefficient, 2.29 × 10^{-5} cm²/s

C_0 - initial concentration of brombenzene, 1.16 × 10^{-6} mol/cm^3

δ - effective diffusion layer thickness, 3.8 × 10^{-3} cm

S1.1.1. Diffusion layer thickness

δ was estimated using particle residence time (t_{res} ≈ 0.72 s) from CFD simulation:

$\delta \approx \sqrt{(2 \times D \times t_{res})}$

$\delta \approx \sqrt{(2 \times 2.29\times10^{-5} \times 0.72)} \approx 3.8 \times 10^{-3}$ cm

S1.1.2. Diffusion coefficient evaluation

For the diffusion coefficient measurements, we used Randles–Sevchik equation using voltammetric curves (CVs) with scan speed variations using Au@Pd (21 nm) deposited on carbon glass electrode[4]. On the CVs, only one cathodic peak was observed at all scanning rates, with no anodic peak (Figure 1), which is confirmed by previous experiments. This indicates that the charge transfer process is not accompanied by significant reverse current and, therefore, is not electrochemically reversible. Additionally, the linear dependence of the peak current i_p/S on the square root of the scanning rate $V^{1/2}$ indicates that the process

proceeds under diffusion control (Figure 2). Thus, the process can be described as diffusion-controlled but kinetically irreversible, which is consistent with the Randles–Shevchik equation for irreversible processes.

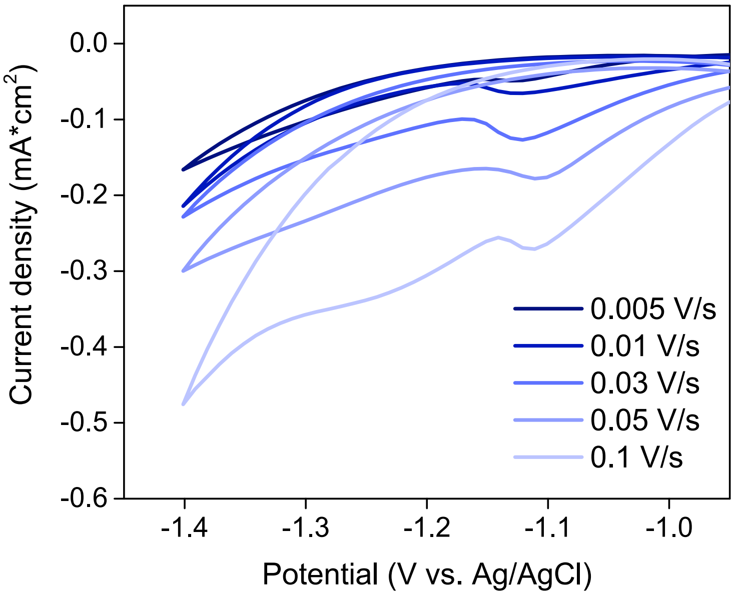

Figure S16. Cyclic voltammograms recorded at different scan rates.

To evaluate the diffusion coefficient of the substance under study, a classical approach based on the Randles–Shevchik equation for an irreversible electrochemical process was used:

$$i_p=(2.69\times10^5)*n*S*\alpha^{1/2}*D^{1/2}*C\cdot V^{1/2}$$

where i_p - peak current (A),

n - number of electrons participating in the electrochemical reaction,

S - electrode area (cm²),

α - charge transfer coefficient,

D -diffusion coefficient (cm²/s),

C - concentration of the substance in the solution (mol/cm³),

V- potential scanning rate (V/s), measurements were taken from low speed to high speed.

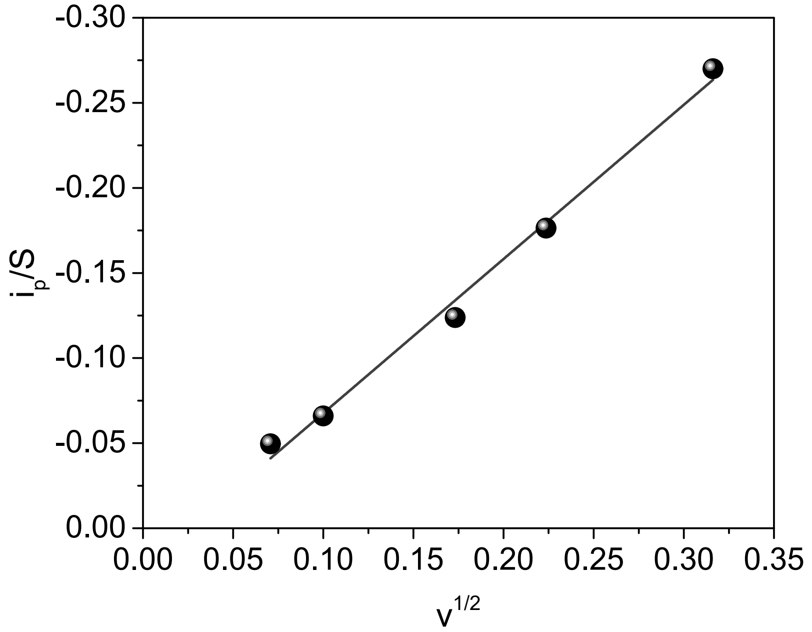

Figure S17. Dependence of peak current on the square root of scan rate.

From the graph of the dependence $i_p/S = \sim V^{1/2}$, the slope of the straight line was obtained, allowing the value of D to be calculated. The charge transfer coefficient $\alpha^{1/2}$ was 0.75. The following values were used in the calculations:

Electrode surface area (S) = 0.196 cm^2.

Br-benz concentration (C) = 1.16×10^{-6} mol/cm^3

Scan speeds (V) = 0.005, 0.01, 0.03, 0.05, 0.1 V/s

Number of electrons (n) = 2

The calculated value of the diffusion coefficient was **D=2.29*10^{-5} cm^2/s**

Plasmon catalytic flux (from kinetic measurements)

The experimentally observed reaction rate under illumination was measured as c = 1.29×10^{-5} s^{-1}/cm^2 (illuminated area A_{illum} is 20.8 cm^2).

Considering catalytic amount of Pd as reaction centers (0.09 wt% loading on 0.5 g) of 3.89×10^{-6} mol, the total reaction rate c_{total} is $c \times A_{illum} \times n_{Pd} \approx 1.04 \times 10^{-7}$ mol/s

The number of Au@Pd per unit area is estimated 2.5×10^{11} NPs/cm^2, and the surface area per NP was calculated as ~1.66×10^{-11} cm^2. This results in an estimated Au@Pd surface area per unit illuminated area of ~4.15 cm^2/cm^2, yielding a total surface area of ~86.3 cm^2 under illumination.

The catalytic flux is defined as the reaction rate per unit of nanoparticle surface area:

$J_{PC} = c_{total} / A_{active} \approx 1.21 \times 10^{-9}$ mol/cm^2/s

Table S3. Parameters used for plasmon catalytic and diffusion flux calculations

Parameter	Value	Unit	Source/Note
Diffusion coefficient (D)	2.29×10^{-5}	cm^2/s	Electrochemical measurements (Randles–Shevchik equation)
Bulk concentration (C_0)	1.16×10^{-3}	mol/L	Reaction condition
Diffusion layer thickness (δ)	3.8×10^{-3}	cm	Estimated from CFD simulations
Catalyst mass	0.95	g	Experimental
Au@Pd loading on Al_2O_3	0.09	wt%	AAS
Total BET surface area	114	cm^2	0.95 g × 120 m^2/g
Turnover number (TON)	833	–	Experimental
Quantum yield (QY)	6.4	%	Experimental
Catalytic rate (c)	1.29×10^{-5}	s^{-1}/cm^2	Kinetic analysis
Total reaction rate (c_{total})	1.04×10^{-7}	mol/s	Calculated: $c \times A_{illum} \times n_{Pd}$
NPs per unit area	2.5×10^{11}	NPs/cm^2	Estimated from size and packing
Surface area per NP	1.66×10^{-11}	cm^2	Calculated from 23 nm diameter
NP surface per cm^2	4.15	cm^2/cm^2	Total NP surface per substrate area
Illuminated area (A_illum)	20.8	cm^2	Experimental setup
Plasmon catalytic flux (J_{PC})	1.21×10^{-9}	$mol/cm^2/s$	c_{total} / A_{active}
Diffusion-limited flux (J_{diff})	2.95×10^{-9}	$mol/cm^2/s$	Fick's law: $D \cdot C_0/δ$

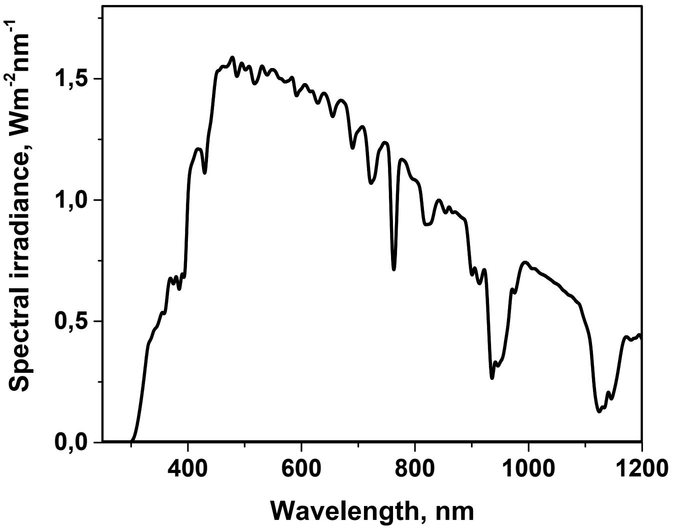

Fig.S18 Solar energy spectral distribution[5]. The figure shows the spectral irradiance of sunlight as a function of wavelength, which serves as a reference for simulating solar light using Xe lamp. The average spectral irradiance of sunlight over the relevant wavelength range was used in the equation for calculating AQY.

References.

1. F. Ehrlich-Sommer, T. Friedl, C. Koller and M.Y.S. Ibrahim, *React. Chem. Eng.*, 2025,**10**, 959-964.
2. C. Batchelor-McAuley, D. Li and R.G. Compton, *ChemElectroChem,* 2020, **7,** 3844-3851.
3. I. Streeter and R.G. Compton, *J. Phys. Chem. C*, 2007, **111**, 18049-18054.
4. Z. Abdi, M. Vandichel, A.S. Sologubenko, M.-G. Willinger, J.-R. Shen, S.I. Allakhverdiev and M.M. Najafpour, *Int. J. Hydrog. Energy*, 2021, **46**, 37774-37781.
5. Lighthouse. Spectrum library. https://www2.pvlighthouse.com.au/resources/optics/spectrum%20library/spectrum%20library.aspx, (accessed September 2025).
